#  Cryptic Prophage Endolysin Is a Highly Active Muramidase

**DOI:** 10.1021/acs.biochem.5c00142

**Published:** 2025-07-09

**Authors:** Per Kristian Thorén Edvardsen, Andrea Nikoline Englund, Åsmund Kjendseth Ro̷hr, Stéphane Mesnage, Gustav Vaaje-Kolstad

**Affiliations:** 1 Faculty of Chemistry, Biotechnology, and Food Science, 393285 Norwegian University of Life Sciences, Ås 1432, Norway; 2 School of Biosciences, University of Sheffield, Sheffield S10 2TN, United Kingdom

## Abstract

Endolysins are phage-encoded enzymes that cleave the peptidoglycan of host bacteria. These enzymes have gained considerable attention due to their ability to cause cell lysis, making them candidates as antibacterial agents. Most genomes, including the common laboratory strains PAO1 and UCBPP-PA14, contain a cryptic prophage encoding a glycoside hydrolase family 19 endolysin (named *Pa*GH19Lys in the present study). Family 19 glycoside hydrolases are known to target peptidoglycan and chitin-type substrates. *Pa*GH19Lys was not active toward chitin but exhibited activity toward chloroform-treated Gram-negative bacteria, displaying ∼10,000-fold higher activity than hen egg white lysozyme. Analysis of products derived from *Pa*GH19Lys activity toward purified peptidoglycan showed that the enzyme catalyzed hydrolysis of the β-1,4 linkage between *N-*acetylmuramic acid and *N-*acetyl-d-glucosamine, classifying the enzyme as a muramidase. Finally, the crystal structure of *Pa*GH19Lys was determined and solved to 1.8 Å resolution. The structure of the enzyme showed a globular α-helical fold possessing a deep but relatively open catalytic cleft.

## Graphical abstract

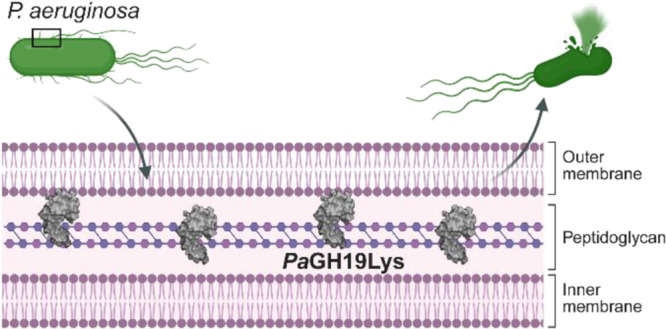

## Introduction

Peptidoglycan (PG) is a bag-shaped macromolecule featuring linear glycan strands cross-linked with short peptides.^1,2^ The polysaccharide part consists of β-1,4-linked *N*-acetyl glucosamine (GlcNAc) and *N-*acetylmuramic acid (MurNAc) as a repeating unit, linked by stem peptides that usually consist of l-Ala-γ-d-Glu-l-X-d-Ala-d-Ala, where X is either the diamino acid mesodiaminopimelic acid (mDAP) or l-lysine.^1–4^ The last amino acid of the stem peptide, d-alanine, is usually lost in the mature PG molecule.^2^ Additional cross-linking of PG chains can occur at positions three or four in the peptide stem.^1,2^ The coupling usually involves the amino group of mDAP or l-lysine, and the carboxyl group of the terminal d-alanine, or through a short lateral peptide bridge in some species.^1^

Enzymatic degradation of PG is achieved through the hydrolysis of either the polysaccharide backbone by glycoside hydrolases or through hydrolysis of the stem peptides by amidases or peptidases.^5^ The glycoside hydrolases (GHs) that cleave the MurNAc-GlcNAc β-1,4 glycosidic bond and generate MurNAc as a reducing sugar are called *N*-acetylmuramidases.^5^ Well-known examples of *N*-acetylmuramidases include lysozyme and mutanolysin, which belong to the glycoside hydrolase (GH) families 22 and 25, respectively,^5–7^ in the carbohydrate active (CAZy) database.^6^ In contrast, enzymes that cleave GlcNAc-MurNAc β-1,4 glycosidic bonds and generate GlcNAc as a reducing sugar are called *N*-acetylglucosaminidases. An example of a known PG hydrolase with *N*-acetylglucosaminidase activity is AtlA from , which belongs to the GH73 family.^8^ PG hydrolases having *N*-acetylmuramidase activity primarily belong to the families GH19, GH22-GH25, GH108, and GH184, while those exhibiting *N*-acetylglucosaminidase activity so far have only been identified in the GH73 family.^6,9^

Family GH19 enzymes were originally believed to occur exclusively in plants and were first discovered as plant pathogenesis-related proteins.^10–13^ These enzymes showed hydrolytic activity toward chitin, a linear polysaccharide of β-1,4-linked *N*-acetyl-d-glucosamine (GlcNAc), and were thus classified as chitinases. It soon became clear that genes encoding GH19 chitinases were also present in bacterial genomes.^6^ The first bacterial GH19 Chitinase, ChiC, from , was described in 1996.^11^ A seminal study by Holm and Sander,^14^ published just a few years prior, had shown that family GH19 chitinases could be classified into a common lysozyme superfamily alongside animal and phage lysozymes, due to their striking structural similarity. This similarity was also observed in a later publication by Wohlkönig et al.^14^ More recently, GH19 proteins from bacteriophages have been found to exhibit activity toward peptidoglycan.^15–18^ This is perhaps not surprising considering the structural similarity of these two β-1,4-linked linear amino-sugar polysaccharides. Presently, the GH19 family contains 16346 entries in the CAZy database, the majority of which are found in bacteria.^6^ The vast majority of characterized GH19 enzymes are chitinases, and the few PG hydrolases that have been biochemically characterized originate from bacteriophages.^6^

In the context of bacteriophages, GH19 PG hydrolases are usually termed endolysins or bacteriophage lysins. These proteins are phage-encoded lytic enzymes that cleave PG in host bacteria.^19^ In combination with holins, small membrane pore-forming proteins, and spanins, proteins that disrupt the outer membrane, endolysins are used by bacteriophages in their late life cycle (lytic cycle) to lyse the bacterium from within.^19–21^ This enables the escape of progeny virions and their subsequent spread. Some bacteriophages may also integrate their genetic material within the bacterial genome, called prophages, and subsequently reproduce with the help of the bacteria.^22^ Prophages can thus remain dormant within the bacterial genome and get activated through environmental stimuli or by regulatory events induced by the bacterium.^23^ In some cases, the prophage genome can be disrupted and fragmented by evolution of the bacterium, leaving intact and functional prophage gene clusters but disabling the ability to form infectious phages. Such gene clusters are called cryptic prophages.^24^ Studies have shown that (cryptic) prophage and phage-like genes can play crucial roles in various bacterial processes, including biofilm formation, toxin secretion, and sporulation.^22,25^ This suggests that some prophage genes can be preserved and functional because they provide a selective advantage to the bacterium, benefiting both the bacterium and the phage.^22,26^ Thus, bacteriophages appear to be a significant factor in the evolution of bacteria through their ability to modify bacterial lifestyle, fitness, and virulence.

Most sequenced genomes, including the common laboratory strains PAO1 and UCBPP-PA14, contain a gene encoding a GH19 endolysin (locus name PA0629/PA14_08160). The gene is located within a cryptic prophage gene cluster^27,28^ that also encodes phage-like pyocins and other phage-related proteins. The pyocins are tail-like bacteriocins that are believed to be important for in pathogenesis, killing of competing bacteria, release of membrane vesicles, and biofilm formation through a lytic release mechanism.^24,29,30^ The mechanism for release is dependent on both the holin proteins (Hol, AlpB, and CidA) and the endolysin Lys of (referred to as *Pa*GH19Lys in the present study). Without these proteins, cell lysis is severely impaired.^24,29^ It is believed that *Pa*GH19Lys is responsible for hydrolyzing the PG polysaccharide, but this has yet to be demonstrated.

In the present study, we show that *Pa*GH19Lys displays *N*-acetylmuramidase activity and can hydrolyze the PG of and other Gram-negative bacteria, supporting the existing hypothesis in the field. The structure of the enzyme, solved to 1.8 Å resolution, shows an α-helical globular fold with a deep and wide active site suitable for accommodating the bulky PG substrate.

## Results

### Analysis of the *Pa*GH19Lys Sequence and Identification of Putative Spanin Proteins

Analysis of the *Pa*GH19Lys ( UCBPP-PA14, PA14_08160) amino acid sequence (UniProt ID: A0A0H2ZLP8) using InterPro^31^ revealed a single-domain protein belonging to the Lysozyme-like domain superfamily. Specifically, the protein is classified into the GH19 family according to both the Pfam^32^ and CAZy databases.^6^ No signal peptide was predicted for the protein using the SignalP 6.0 server,^33^ indicating that the protein is produced as a functional enzyme when translated in the cytoplasm. Furthermore, the protein was found in 297 strains,^34^ but also in several other *Pseudomonas* species, suggesting that the protein is prevalent in both and in the *Pseudomonas* genus in general.

As previously described, other studies have noted that the *Pa*GH19Lys encoding gene *lys* is located in a large cryptic prophage gene cluster (Figure 1).^24,30,35^ Inspection of the gene neighborhood in UCBPP-PA14 showed that *lys* (PA14_08160) exists in an operon with two other genes annotated as hypothetical proteins (PA14_08180 and PA14_08190) (Figure 1). Further insight into the putative functions of these proteins could provide a better understanding of the role of *Pa*GH19Lys, and thus, their sequences were analyzed using a bioinformatic approach. Sequence analysis using InterPro and SignalP v6.0,^31,33^ did not associate the proteins with any known protein families, but indicated that both proteins contain signal peptides. More specifically, SignalP v6.0 predicted that PA14_08190 contains a lipoprotein signal peptide (Sec/SPII), suggesting lipidation due to cysteine in +1 of the cleavage site.^33^ The lytic cycle of bacteriophages involves membrane-accumulating lipoproteins called “spanins” that are expressed together with holin and endolysin proteins.^36–39^ Based on this knowledge, it is reasonable to hypothesize that PA14_08180 and PA14_08190 encode spanins. To investigate this hypothesis, the two protein sequences were aligned with the LysB and LysC spanins of bacteriophage P2. The analysis showed that the PA14_08180 and PA14_08190 sequences were 22.97 and 18.75% identical to LysB and LysC, respectively (Figure S1A,B). The predicted Alphafold structure of PA14_08180 appears to adopt an alpha helical domain, in line with a previous study on phage spanins.^21^ Furthermore, PA14_08190 contains eight prolines and four cysteines, most of which are preserved compared to LysC, and is a characteristic of the so-called *o*-spanins (Figure S1B).^21^ In summary, based on the data presented herein, we propose that PA14_08180 and PA14_08190 most likely encode putative spanin proteins that are possibly coexpressed with *Pa*GH19Lys, given their presence in an operon.

**Figure 1:**
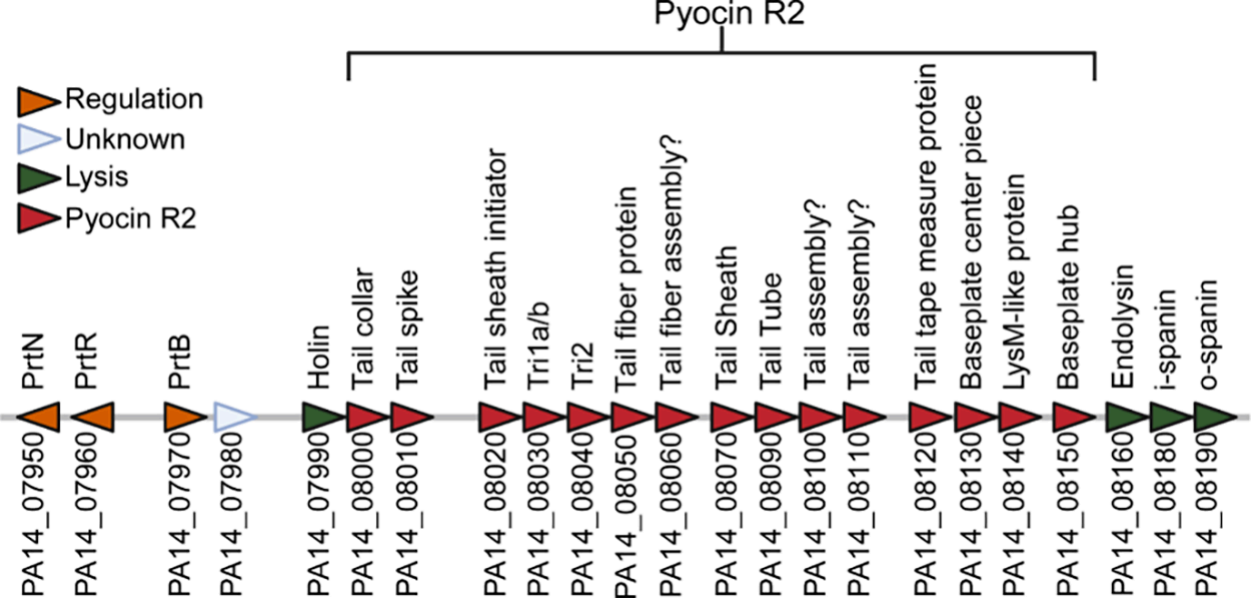
Genetic organization of the R2 pyocin gene cluster together with the regulatory and lysis genes of the cryptic prophage cluster of UCBPP-PA14. The genetic loci and encoded proteins are shown.

### Expression and Purification of *Pa*GH19Lys

The gene encoding *Pa*GH19Lys was amplified by PCR and cloned into the pET-28a vector via the *NcoI* and *XhoI* restriction sites. The recombinant His-tagged protein was expressed in strain C43 as a soluble cytoplasmic protein. Purification was performed using immobilized metal affinity chromatography followed by hydrophobic interaction chromatography. Typical yields of purified protein ranged from 3 to 8 mg per liter of culture. SDS-PAGE analysis revealed a single band at ∼25 kDa (Figure S2), compatible with the predicted molecular weight of 23 kDa for *Pa*GH19Lys.

### Crystal Structure of *Pa*GH19Lys

*Pa*GH19Lys was successfully crystallized with a C-terminal hexahistidine tag and solved to a resolution of 1.8 Å (PDB accession number: 9EOI) using the predicted AlphaFold2 model of the protein (Alphafold database ID: AF-A0A0H2ZLP8-F1) as a molecular replacement template (Figure 2A). The *Pa*GH19Lys structure displayed a fold consisting of α-helixes. The helices are packed to form a structure with two subdomains, the large subdomain (Met1-Leu55, Pro155-Ser209) and the small subdomain (Thr56-Gln154) (Figure 2A). The protein contains a deep-spanning catalytic cleft with an active site comprised of amino acids H50, E51, E60, Y103, Q127, T129, I187, N188, and R197 (Figure 2B). Among these, E51 and E60 aligned structurally with the catalytic base and acid glutamates in the structurally and biochemically characterized GH19 chitinases (Figure 2B),^17^ suggesting similar roles in *Pa*GH19Lys.

**Figure 2:**
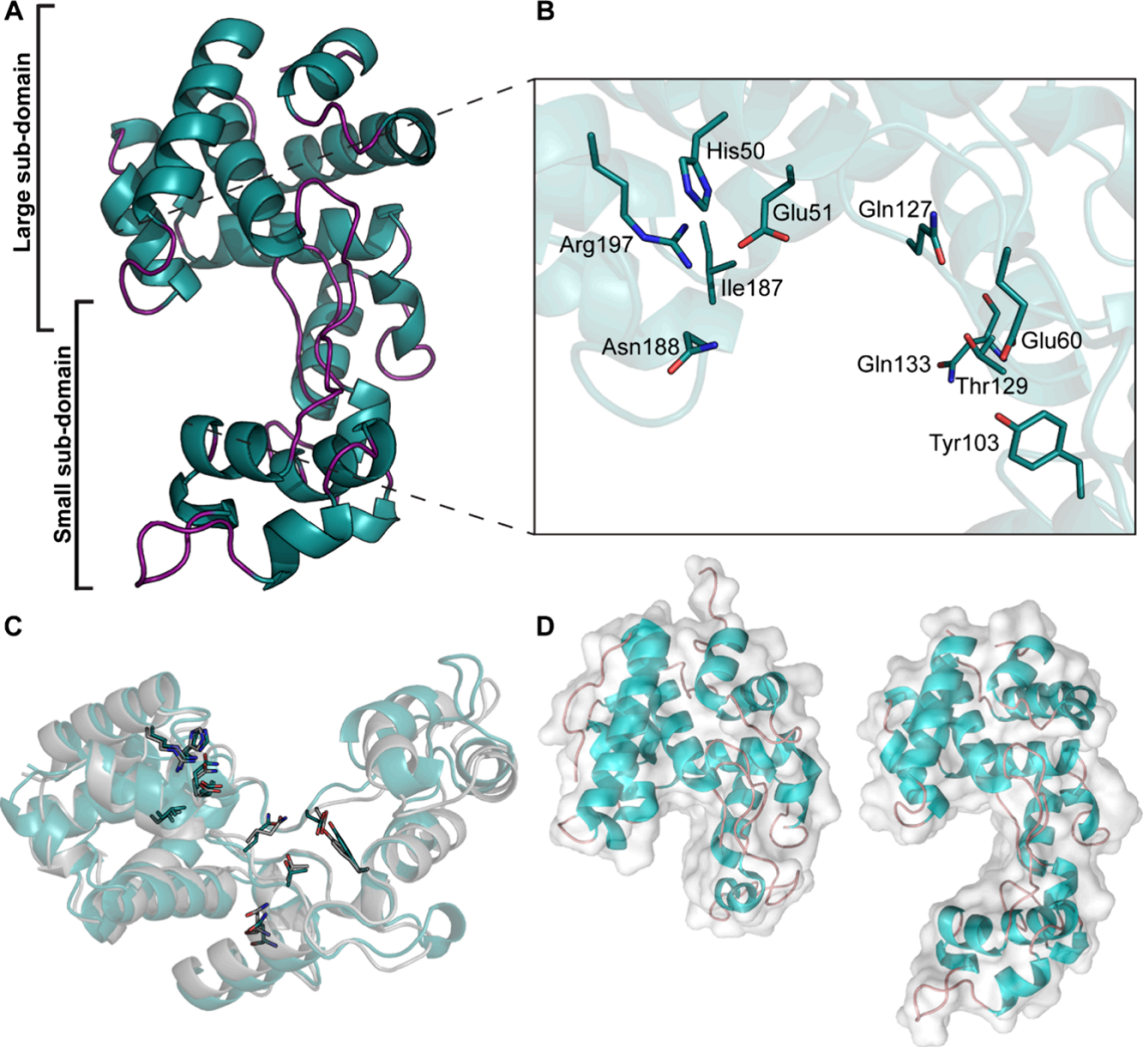
Crystal structure overview of the full-length *Pa*GH19Lys protein and active site. (A) The crystal structure of *Pa*GH19Lys is shown as a cartoon representation, (B) with an inset showing the highly conserved amino acids in the active site. (C) Comparison of the structures and active sites of *Pa*GH19Lys (blue-green) with the bacteriophage SPN1S endolysin (gray) (PDB accession number: 4OK7). (D) Comparison of the structures of the GH19 domains of *Sc*ChiC from *Streptomyces* (PDB accession number: 1WVU) (left) and *Pa*GH19Lys (PDB accession number: 9EOI) (right). The GH19 domains are shown in a combination of cartoon and (transparent) surface representation.

By searching the Dali server,^40^ the endolysin from the *Salmonella* bacteriophage SPN1S (PDB accession number: 4OK7) was identified as the structure most similar to *Pa*GH19Lys, yielding an α-carbon RMSD of 1.6 Å and a Z-score of 25.8. The SPN1S endolysin has been shown to efficiently lyse Gram-negative bacteria.^18^ Comparison of the structures of *Pa*GH19Lys and the SPN1S endolysin revealed substantial similarities, particularly in the active sites (Figure 2C), suggesting that they have similar functions.

Structural comparison of the GH19 endolysin *Pa*GH19Lys with the well-characterized GH19 Chitinase *Sc*ChiC revealed that the catalytic cleft of the former is considerably larger than that of the latter, due to a large insertion that forms a deeper, but also more spacious cleft (Figure 2D). The topology of the endolysin active-site cleft may reflect adaptation to accommodate the peptide stems of the PG.

### *Pa*GH19Lys Does Not Hydrolyze Chitin or Chitosan

As the GH19 family of proteins contains enzymes with activity against chitin, chitosan, PG, or all three,^17^ the activity of *Pa*GH19Lys toward the two former polysaccharides was investigated. No products were observed by HPLC analysis when incubated with α-chitin, β-chitin, chitohexaose, or chitosan (Figure S3A–F). These results indicate that *Pa*GH19Lys does not possess chitinolytic activity. This observation is consistent with the broader trend observed among enzymes of the lysozyme superfamily fold (GH19, GH22, GH23, GH24, and GH46), wherein some enzymes are strictly active toward peptidoglycan, whereas others are more promiscuous. For example, peptidoglycan lyases typically lack activity toward chitin, whereas several lysozymes exhibit catalytic activity on both peptidoglycan and chitin substrates.^14^

### Lytic Activity and Biochemical Characterization

To determine the potential lytic activity of *Pa*GH19Lys, chloroform-treated cells were incubated with different concentrations of the protein, including hen egg-white lysozyme, as a positive control. The chloroform treatment weakens the outer membrane of Gram-negative bacteria, rendering them sensitive to PG-degrading enzymes, while maintaining the integrity of the cell wall sacculi. In the presence of *Pa*GH19Lys, a reduction in the turbidity of chloroform-treated was observed, demonstrating the lytic activity of the protein (Figure 3A). Once the activity of *Pa*GH19Lys had been verified, the optimum pH and temperature for the lytic activity were determined. By monitoring the linear decline in OD450 (ΔOD450) in the first minute of the initial reaction and calculating the slope of the curve, we determined the initial lytic activity of the enzyme under the different conditions tested. The enzyme showed a typical bell-shaped pH-dependent activity profile, with maximum activity observed in the pH 7–8 range (Figure 3B), and the optimal activity of the enzyme was determined to be 37 °C (Figure 3C). Activity was also observed at 4 °C (Figure S4). Furthermore, when chloroform-treated cells were incubated with different concentrations of *Pa*GH19Lys, a reduction in turbidity was observed in a concentration-dependent manner (Figure 3D). The turbidity rapidly declined within 5 min of incubation with the enzyme, even with as little as 0.1 nM *Pa*GH19Lys (Figure 3A). Interestingly, the lowest concentration of *Pa*GH19Lys tested (0.1 nM) was still substantially more lytic than the 1.0 μM lysozyme, suggesting that the endolysin is highly potent.

**Figure 3:**
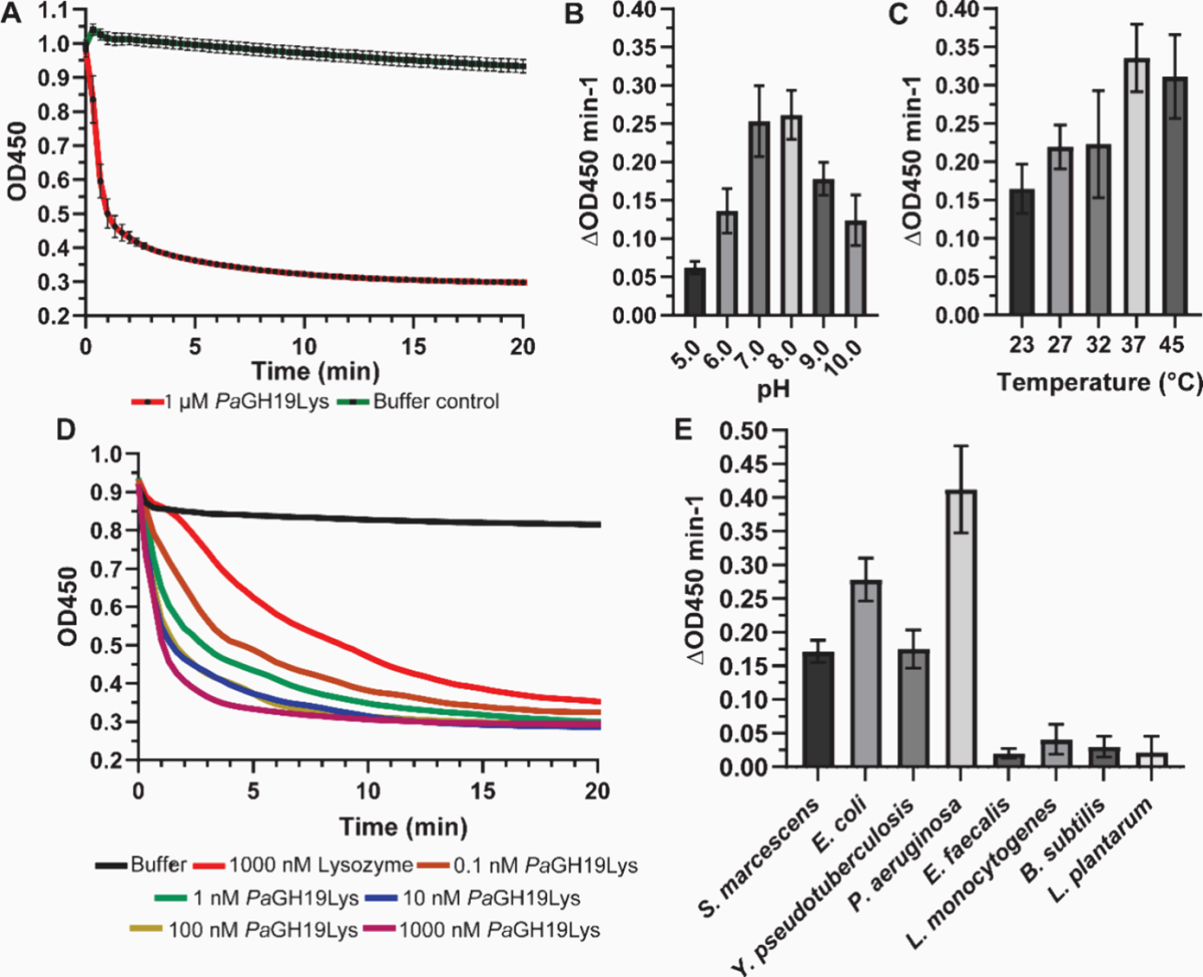
Lytic activity and physiochemical properties of *Pa*GH19Lys. (A) Activity of *Pa*GH19Lys against chloroform-treated . Activity of *Pa*GH19Lys with varying (B) pH (using the universal Britton–Robinson buffer) and (C) temperature levels, using sacculi as a substrate. (D) Chloroform-treated incubated with various concentrations of enzyme. (E) Lytic activity of *Pa*GH19Lys against Gram-negative and Gram-positive bacteria. Unless stated otherwise, 1 μM *Pa*GH19Lys was used for all reactions. The data are plotted as the mean ± standard deviation when indicated, representing four independent experiments. Lytic activity was determined by calculating the decrease in OD450 during the first minute of the reaction using linear regression.

We next investigated the lytic action of the enzyme toward other Gram-negative and Gram-positive bacteria. In a 20 min endpoint assay, *Pa*GH19Lys displayed clear activity against chloroform-treated Gram-negative bacteria such as , , and , but lower activity than for (Figure 3E). Essentially no activity of the endolysin was observed using intact cells of Gram-positive bacteria such as , , , and as substrate (Figure 3E).

### Identification of the Catalytic Residues of *Pa*GH19Lys

Based on the structural comparison of *Pa*GH19Lys with the well-characterized GH19 endolysin SPN1S (Figure 2C),^18,41^ the amino acids E60 and E51 were identified as the putative catalytic acid and base, respectively. To determine the importance of E60 in catalysis, the *Pa*GH19Lys_E60Q_ variant was constructed, expressed, purified, and characterized using a lysis assay. At 1 nM, the enzymatic activity of *Pa*GH19Lys_E60Q_ was not observable (Figure 4A). Some lytic activity could be observed for the E60Q variant at 1 μM (Figure 4B), but it was still significantly lower than that of the wild-type protein (Figure 4B). These data support the idea that E60 is important for catalysis by *Pa*GH19Lys.

**Figure 4:**
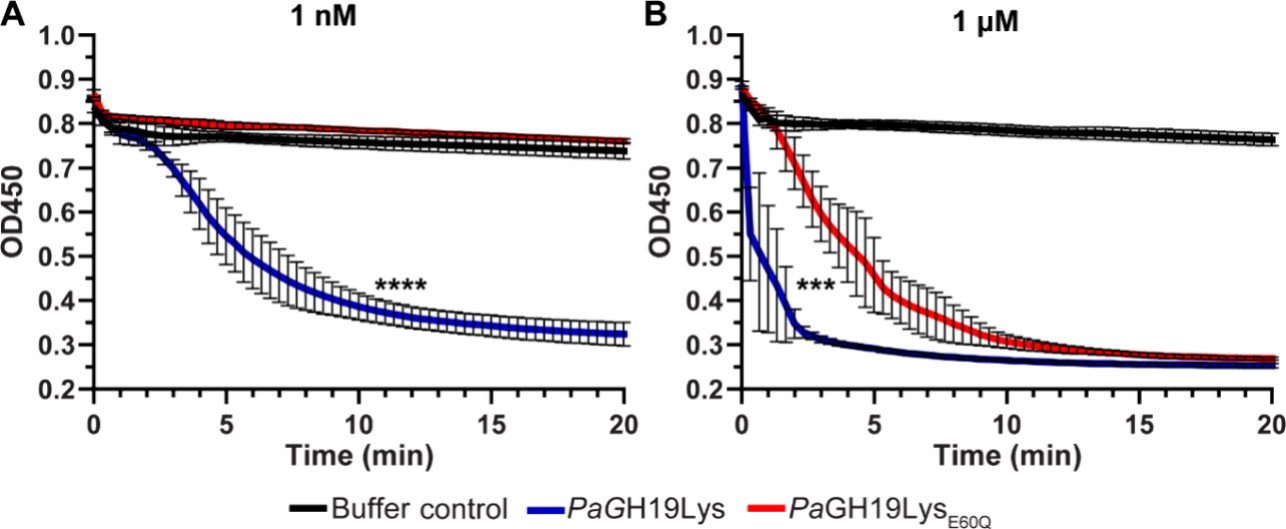
Lytic activity of the wild-type *Pa*GH19Lys and the *Pa*GH19Lys_E60Q_ variant. Lytic activity toward chloroform-treated cells was monitored at 37 °C, pH 8.0, using two different concentrations of the enzyme with (A) 1 nM and (B) 1 μM of both *Pa*GH19Lys and *Pa*GH19Lys_E60Q_. *** *p* < 0.001 and **** *p* < 0.0001; *Pa*GH19Lys versus *Pa*GH19Lys_E60Q_ by two-tailed unpaired Welch’s *t* test. All data are presented as the mean ± standard deviation, representing four independent replicates.

Based on a previous study that analyzed conserved amino acids in the catalytic domains of the GH19 family, including both endolysins and chitinases, the conserved amino acid corresponding to E51 in *Pa*GH19Lys was hypothesized to be the catalytic base.^17^ A study by Turnbull et al.^24^ demonstrated that a *lys* (PA0629) mutant encoding an E51 V substitution in PAO1 was unable to cause lysis, supporting the hypothesis that this residue is important for the activity of the protein. Unfortunately, despite several attempts, the production and purification of the *Pa*GH19Lys_E51Q_ variant were not accomplished due to difficulties in protein expression.

### Determination of Glycosidic Bond Specificity in Peptidoglycan Hydrolysis

To determine the specificity of *Pa*GH19Lys, the reaction products from the hydrolysis of purified PG from were analyzed using UPLC-MS after the chemical reduction of the reducing ends (Figure S5A-F). The dominating product showed two major ions with *m*/*z* values at 942.41 and 471.71, corresponding to the single or double proton adduct, respectively, of the disaccharide tetrapeptide GlcNAc-MurNAc-l-Ala-Glu-mDAP-Ala (Figure 5A). An identical (dominant) product was observed when using *N*-acetylmuramidase mutanolysin as a positive control (Figure 5B). Since the product had been chemically reduced, analysis of the fragmentation pattern of the analyte could be used to determine the identity of the reducing end monosaccharide, as described by Eckert et al.,^8^ the masses of the diagnostic fragments expected from *N*-acetylmuramidase and *N*-acetylglucosaminidase activity are 739.32 or 759.35, respectively, representing either a native or chemically reduced GlcNAc^8^ (Figure 5C,D). The mass of the diagnostic fragment observed in this study was 739.32 (Figure 5A), demonstrating that *Pa*GH19Lys is an *N*-acetylmuramidase, similar to mutanolysin.

**Figure 5:**
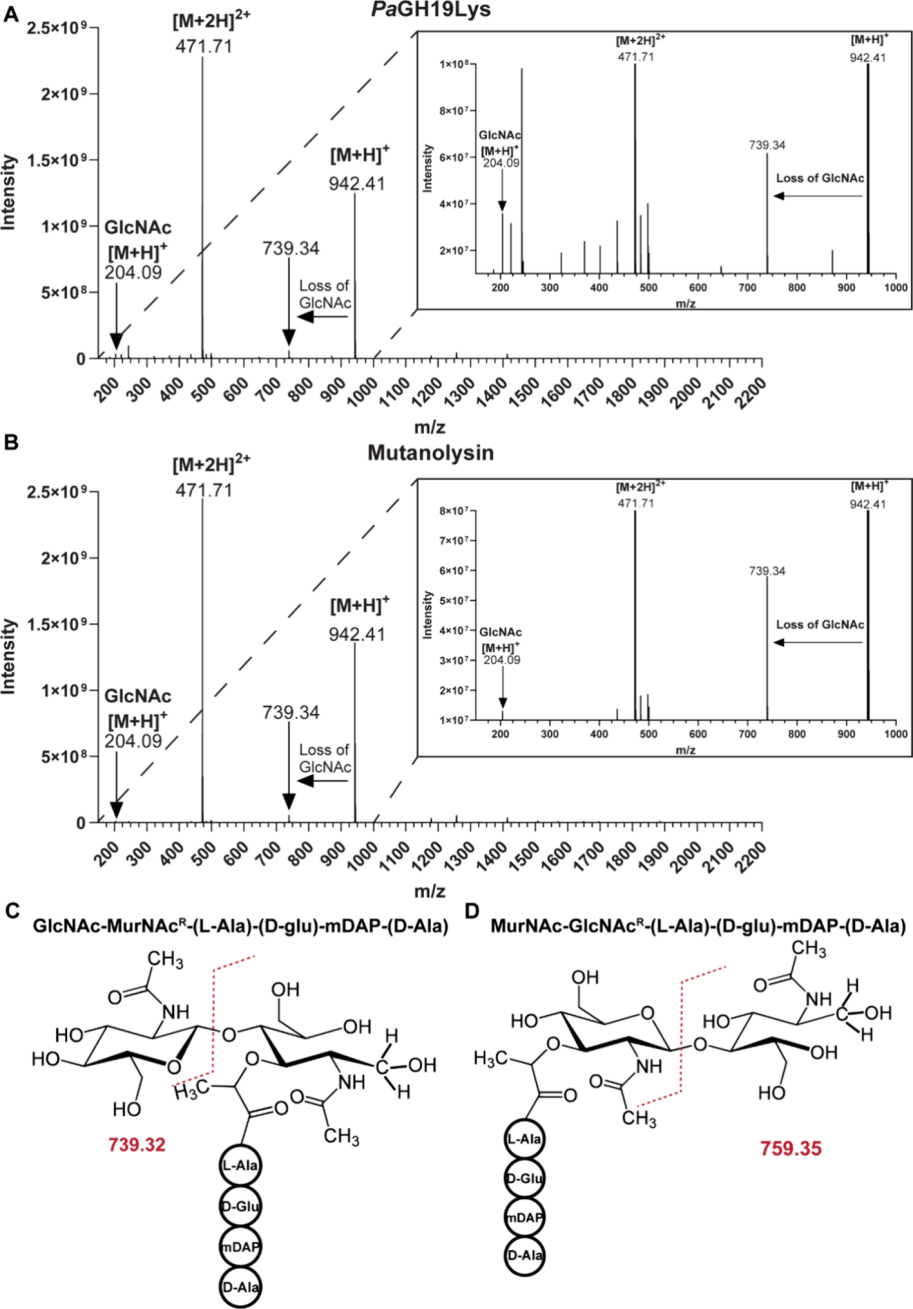
Determination of the hydrolytic specificity of *Pa*GH19Lys by UPLC-MS. Products arising from PG hydrolysis by (A) *Pa*GH19Lys and (B) mutanolysin are shown as MS spectra with in-source fragmentation. To allow examination of specific features in more detail, zoomed-in portions of the spectra are shown as inset panels in the top right corner. The reaction mixtures were incubated for 24 h at 37 °C, prior to chemical product reduction, to enable the analysis of endpoint products. Panels (C) and (D) show the fragmentation (indicated by the dashed red line) and *m*/*z* of the PG monomer resulting from *N-*acetylmuramidase or *N-*acetylglucosaminidase activity, respectively.

### Analysis of Products Obtained from Enzymatic Peptidoglycan Hydrolysis

A comparative analysis of the enzymatic activity of *Pa*GH19Lys and mutanolysin was performed to assess their enzymatic activity, aiming to determine whether *Pa*GH19Lys produced any atypical products or whether the enzyme needed a specific motif or modification of PG to carry out its catalytic function. The PGfinder software^42^ was used to examine the PG monomer (i.e., disaccharide peptides of PG) product profiles for the two enzymes (Table S1; see Figures S6–S22 for representative spectra). Additionally, searches were performed for PG monomers with deacetylated, acetylated, or anhydro-MurNAc sugar products (Table S1). The UPLC-MS data analysis of reaction products arising from a 24h PG-digest at 37 °C revealed a high similarity between independent replicates, indicating that the quantification was robust and reproducible (Figure 6A; Figure S5A–F). The analysis indicated that *Pa*GH19Lys and mutanolysin had comparable product profiles (Figure 6B,C), differing only in the presence of gm-A and gm-AEJT for mutanolysin and gm-AEJAK for *Pa*GH19Lys (Table S1). As these products could not be verified by MS2, they were omitted from the analysis. Additionally, modified PG monomers were detected for both enzymes based on MS1 data (Table S1), but their specific modifications could not be identified through MS2 data analysis using the proprietary Protein Metrics Byos software. The modifications searched for using PGfinder were present in both product profiles for the enzymes, indicating that none of the modifications were exclusively detected for either enzyme (Table S1). Finally, to determine whether there were statistically significant differences in the abundance of PG monomers present in all replicates for both enzymes, potentially indicating substrate-binding preference, the fold change of individual PG monomers was analyzed by comparing *Pa*GH19Lys with mutanolysin. (Figure 6D). The volcano plot showed no significant difference in the quantity of products released when comparing *Pa*GH19Lys and mutanolysin (Figure 6D). These results suggest that the two enzymes act similarly on PG, despite representing two different GH families and their origins.

**Figure 6:**
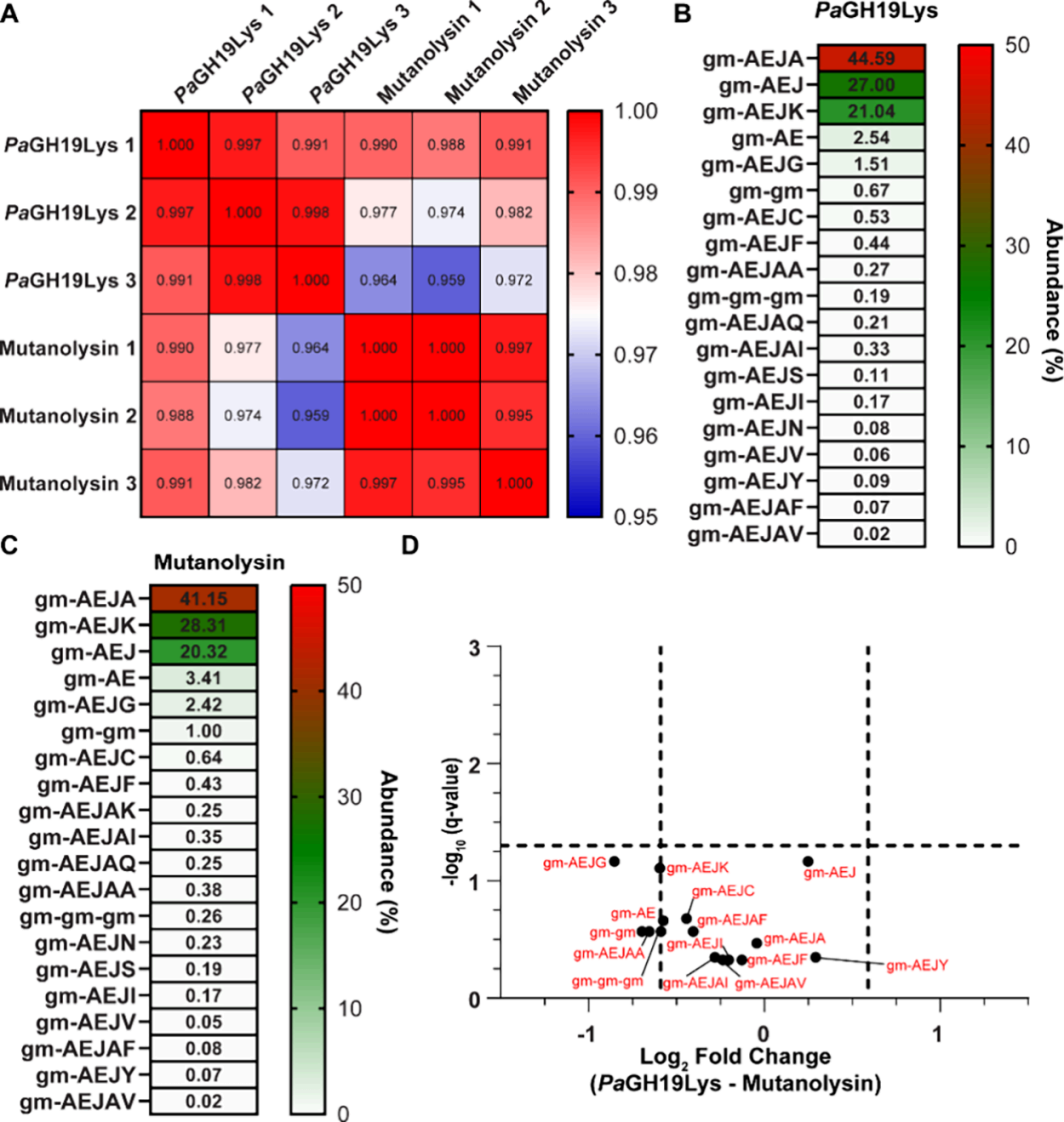
PG product analysis was by UPLC-MS. (A) Heatmap showing the Pearson’s correlation coefficients across the three reactions replicates for *Pa*GH19Lys and mutanolysin. The PG monomeric product profiles for (B) *Pa*GH19Lys and (C) mutanolysin showing the average relative abundances of the different structures represented by heatmaps. (D) Volcano plots displaying the log2 fold change of each detected monomeric PG structure and their corresponding *p*-values (−log10) within the data set. The plot compares the PG structures detected for *Pa*GH19Lys with those identified for mutanolysin. Significance was determined by an unpaired two-tailed *t* test, and the cutoff was defined as *p* = 0.05 (−log10 = 1.3) and (±) 1.5-fold change (log2 = 0.58). All data were plotted as the mean of the triplicate reactions.

## Discussion

Bacteriophages require PG-hydrolyzing enzymes to enable lysis of the bacterial host cell and release of the newly formed viral particles.^39^ Interestingly, cryptic prophage genes seem to be harnessed by a variety of bacteria to improve fitness; for example, by inducing altruistic cell lysis for protein secretion or release of eDNA.^22,24,29,43^ This is indeed the case for *,* ^24,29^ for which the majority of the sequenced strains contain cryptic prophage genes related to cell lysis. One of these genes is *lys*, which encodes the family GH19 *Pa*GH19Lys. Although this protein has been shown to lyse cells,^24,29,35^ little is known about the biochemical properties of the enzyme. In the present study, we provide evidence that *Pa*GH19Lys is a muramidase, given its hydrolysis of the MurNAc-GlcNAc glycosidic bond (Figure 5A–D). Although such property could be expected from comparison with an orthologous enzyme from a bacteriophage targeting *Salmonella* Typhimurium,^18,41^ none have hitherto performed such detailed analysis of *Pa*GH19Lys. Analysis of products released by *Pa*GH19Lys from hydrolysis of UCBPP-PA14 PG showed dominance of the AEJA, AEJ, and AEJK stem peptides (Figure 6B). These three stem peptides may reflect the general composition of PG. Indeed, previous studies have also shown the composition of the PG of to be composed mainly of the AEJA, AEJ, and AEJK stem peptides.^44–47^ Also, it would be expected that *Pa*GH19Lys and mutanolysin have different substrate specificities given their difference in sequence and structure; however, both enzymes showed comparable product profiles (Figure 6B,C).

A noteworthy observation from the PG-degradation product analysis was the considerable diversity observed in the fourth amino acid of the stem peptides (Figure 6B,C). This diversity may be attributed to the anchoring of PG to lipoproteins, such as Braun’s lipoprotein, which is known to link PG to the outer membrane and provide stability to the envelope.^47–50^ Apart from Braun’s lipoprotein, other proteins have also been reported to be tethered to PG.^51,52^ Another, perhaps more plausible, explanation is that the diversity is a consequence of the L, D-transpeptidation exchange.^50,53^

*Pa*GH19Lys demonstrated high catalytic activity, surpassing lysozyme in its ability to lyse chloroform-treated , even at a concentration that was 10,000 times lower (Figure 3D). Compared with other endolysins, *Pa*GH19Lys appears to be a potent endolysin, even at low enzyme concentrations.^18,54–57^ Although not as active against other bacteria, *Pa*GH19Lys could be a promising candidate as a possible enzyme-based antibacterial agent against in combination with an outer membrane permeabilizing agent, owing to its potency and specificity. The high specificity of the enzyme toward likely reflects its biological function within the bacterium, and it may exhibit reduced specificity against other bacteria due to differences in their PG structure.^2^

The present findings indicate that the cryptic prophage endolysin *Pa*GH19Lys, encoded in the R- and F-pyocin gene cluster of , is a muramidase responsible for the lysis of cells, as previously suggested.^24,29,35^ Indeed, a study by Turnbull et al.^24^ found that the mRNA transcript levels of R- and F-pyocin gene clusters were significantly higher in isolated membrane vesicles produced upon lysis than in stationary phase cells. Additionally, the release of eDNA in biofilms is impaired when the genes *alpB* and *hol*, which encode for holin proteins, and *lys* are deleted from the genome.^29^ Our results support the proposed model, whereby uses holin proteins (Hol, AlpB, and CidA) in combination with the cryptic prophage *Pa*GH19Lys endolysin^24,29^ to induce bacterial lysis, which is crucial for membrane vesicle production, pyocin release, and biofilm formation. However, further studies are necessary to determine the role of the putative spanin proteins (Figure S1A,B) in the lysis process.

## Conclusions

Collectively, our findings clarify the biochemical properties, structural organization, and biological relevance of *Pa*GH19Lys, a cryptic prophage-derived endolysin from . We establish that *Pa*GH19Lys is a highly active muramidase that efficiently lyses peptidoglycan. The crystal structure reveals an α-helical fold with a spacious catalytic cleft tailored for accommodating the peptidoglycan. Taken together, these results suggest that *Pa*GH19Lys participates in native cell lysis processes, such as membrane vesicle release and biofilm development. The reported data underscore the promise of *Pa*GH19Lys as a scaffold for future anti- therapeutics.

## Materials and Methods

### Primers and Bacterial Strains

The primers and bacterial strains used in this study are given in Tables 1 and 2 [Table tbl2]

**Table 1:** Description and Summary of the Primers Used in This Study

**primers**	**sequence**	**ref**
GH19 PA14 pET-28a forward NcoI	AGGAGATATACCATGGGGATGAAACTGACCGAACAG	this study
GH19 PA14 pET-28a reverse XhoI	GGTGGTGGTGCTCGAGGCTCAGAACGGCAC	this study
pNIC forward	TGTGAGCGGATAACAATTCC	this study
pNIC reverse	AGCAGCCAACTCAGCTTCC	this study
GH19 PA14 pET-28a E51Q forward	GCACAGGTTGGTCATCAAAGCAGCCAGCTGA	this study
GH19 PA14 pET-28a E51Q reverse	TCAGCTGGCTGCTTTGATGACCAACCTGTGC	this study
GH19 PA14 pET-28a E60Q forward	GCTGACCCGTCTGGTGCAAAATCTGAATTATAGC	this study
GH19 PA14 pET-28a E60Q reverse	GCTATAATTCAGATTTTGCACCAGACGGGTCAGC	this study

**Table 2:** Summary of the Bacterial Strains Used in This Study

**strain**	**Reference**
UCBPP-PA14	^58^
BJL200	^59^
One Shot BL21 Star (DE3)	purchased from Invitrogen
EGD	^60^
V583	^61^
FH-Ba-0594	gift from the Norwegian Institute of Public Health
WCFS1	^62^
WB800N	purchased from MoBiTec GmbH
One Shot BL21 Star (DE3) pET-28a *Pa*GH19Lys (PA14_08160) with His tag	this study
One Shot BL21 Star (DE3) pET-28a *Pa*GH19Lys E51Q (PA14_08160) with His tag	this study
One Shot BL21 Star (DE3) pET-28a *Pa*GH19Lys E60Q (PA14_08160) with His tag	this study
C43 (DE3) pET-28a *Pa*GH19Lys (PA14_08160) with His tag	this study
C43 (DE3) pET-28a *Pa*GH19Lys E51Q (PA14_08160) with His tag	this study
C43 (DE3) pET-28a *Pa*GH19Lys E60Q (PA14_08160) with His tag	this study

### Cloning

The lytic enzyme (PA14_08160) (UniProt ID; A0A0H2ZLP8) of UCBPP-PA14 was synthesized and codon optimized for by Thermo Fisher Scientific using their gene synthesis service. Thereafter, the gene was amplified by PCR using the primers GH19_PA14_pET28_Forward_NcoI and GH19_PA14_pET28_Revers_XhoI, and then cloned in pET-28a using the In-Fusion HD cloning kit (Clontech). The final construct of pET-28a with the codon-optimized insert of PA14_08160 was transformed and propagated in One Shot BL21 Star (DE3) (Invitrogen) and OverExpress C43 (DE3) (Sigma) cells. Truncated versions of the lytic enzyme were generated using the QuikChange II XL Site-Directed Mutagenesis Kit according to the manufacturer’s instructions. The predicted catalytic residues E51 and E60 were mutated to glutamine residues E51Q and E60Q, respectively. All constructs were verified by sequencing.

### Expression and Purification of *Pa*GH19Lys and *Pa*GH19Lys_E60Q_ from C43

Expression of His-tagged and the truncated versions was performed by the cultivation of C43 containing the relevant plasmid in Terrific Broth (TB) medium supplemented with 50 μg/mL kanamycin. Isopropyl β-d-1-thiogalactopyranoside (IPTG) was added to a final concentration of 0.2 mM when the culture reached OD600 = 0.6–0.8. The culture was further incubated at 37 °C for an additional 3 h before the pellets were harvested. The bacterial cells were harvested by centrifugation at 14,000*g* for 15 min and resuspended in lysis/binding buffer (5 mM imidazole, 150 mM NaCl and 15 mM Tris-HCl pH 7.5 supplemented with Complete Mini EDTA free protease inhibitors with a final concentration of 1× and a cocktail of phosphatase inhibitors: 1 mM beta-glycerophosphate (Sigma), 1 mM sodium orthovanadate (Sigma), 10 mM sodium pyrophosphate (Sigma) followed by sonication using a Vibra Cell ultrasonic processor (Sonics). The cells were sonicated for 10 min using a cycle of 5 s off and 5 s on (30% amplitude). Cell debris was removed by centrifugation at 48,000*g* for 15 min, and the cytoplasmic protein extract was filtered using a 0.2 μm filter.

Cytoplasmic extracts were loaded onto a HisTrap high-performance column (Cytiva/GE Healthcare) connected to a KTA pure protein purification system (Cytiva/GE Healthcare), and purification was performed based on the manufacturer’s instructions.

Unwanted proteins were removed by hydrophobic interaction chromatography using a KTA pure (Cytiva/GE Healthcare) operating a HiTrap Phenyl HP column (Cytiva/GE Healthcare). Purification was performed based on the manufacturer’s instructions with the following buffers: 20 mM Tris-HCl pH 7.5 with 1 M NH_4_SO_4_ (buffer A) and 20 mM Tris-HCl pH 7.5 (buffer B).

Fractions were pooled, concentrated, and buffer exchanged into 20 mM Tris-HCl pH 7.5, using Vivaspin 20 (10-kDa molecular weight cutoff) centrifugal concentrators (Sartorius Stedim Biotech GmbH). Protein purity was estimated using SDS-PAGE to be >90%. Protein concentrations were determined using the theoretical extinction coefficient calculated by the ProtParam tool (http://web.expasy.org/protparam/) at absorbance 280 nm.

### *Pa*GH19Lys Activity against Chitin, Chitosan, and Chitohexaose

The reaction mixtures (200 μL) contained 50 mM Tris-HCl buffer pH 8.0 and 1 μM of *Pa*GH19Lys () with α-chitin (final concentration of 5 g/L), β-chitin (final concentration of 5 g/L), chitosan, or (GlcNAc)_6_ (final concentration of 0.1 g/L) as substrate. The reactions were incubated at 37 °C for 2 h with shaking at 600 rpm in a Thermomixer C (Eppendorf, Hamburg, Germany). After incubation, the samples were centrifuged, stopped by adding H_2_SO_4_ to a final concentration of 5 mM, and centrifuged at 16,900*g* for 5 min in an Eppendorf 5418R centrifuge. The supernatant was obtained and then filtered using a MultiScreenHTS HV Filter Plate of 0.45 μm (Millipore). Degradation products were analyzed by a Dionex Ultimate 3000 HPLC system using UV detection at 194 nm for detection of the analyte. The samples were analyzed using the HPLC system with a 100 × 7.8 mm Rezex RFQ-Fast Acid H+ (8%) analytical column (Phenomenex, Torrance, CA, USA) at 85 °C with 5 mM H_2_SO_4_ as the eluent, using an isocratic flow of 1.0 mL/min. The injection volume was set to 8 μL.

### Preparation of Bacteria for Lytic Activity Assay

UCBPP-PA14 was grown overnight in LB. The next day, the culture was diluted 1:50 in LB and grown for 6 h (mid-exponential phase) at 37 °C with shaking (200 rpm). The bacterial cultures were harvested using a centrifuge at 4700*g* for 10 min. Thereafter, the pellet was washed once with 50 mM Tris-HCl pH 8.0 before being treated with Tris-saturated chloroform for 45 min at room temperature with rotation using a multirotator RS-60 (Biosan) to perforate and partially remove the outer membrane. Following the chloroform treatment, the bacteria were washed three times with 50 mM Tris-HCl, pH 8.0, in order to fully remove the chloroform. Bacteria were either used directly in lysis assays or frozen at −80 degrees for storage until use.

To investigate the lytic activity of *Pa*GH19Lys across Gram-negative and positive bacteria, UCBPP-PA14, BJL200, One Shot BL21 Star (DE3), EGD, V583, FH-Ba-0594, WCFS1, and WN800N were prepared for use in lytic assays. First, individual cultures of all bacteria were grown overnight in BHI, a medium that is equally suitable for Gram-negative and positive bacteria. The next day, the bacteria were diluted 1:50 in BHI and grown for 6 h at 37 °C with shaking (200 rpm). The bacterial cultures were harvested using a centrifuge at 4700*g* for 10 min. Thereafter, the pellets for the Gram-negative bacteria were washed once with 50 mM Tris-HCl pH 8.0 before being treated with Tris-saturated chloroform for 45 min at room temperature with rotation using a multirotator RS-60 (Biosan). Following the chloroform treatment, the bacteria were washed three times with 50 mM Tris-HCl, pH 8.0, in order to remove the chloroform fully. On the other hand, the Gram-positive bacteria were not treated with chloroform and only washed with 50 mM Tris-HCl, pH 8.0. All bacterial substrates were either used directly in assays or frozen at −80 °C until use.

### Lytic Activity Assay

The lytic activity of *Pa*GH19Lys, or the *Pa*GH19Lys_E60Q_ variant, was determined by monitoring the lysis of chloroform- or buffer-treated bacterial suspensions by the enzyme in a 96-well format by using a Varioskan LUX multimode microplate reader (Thermo Fisher Scientific). Lysis was determined by monitored decline in OD450 at 10 s intervals over a time span of 20 min. The initial activity of a lytic reaction was calculated as the slope of the linear decrease in OD450 during the first minute of the reaction, using linear regression. All reactions were performed in 50 mM Tris-HCl pH 8.0 at 37 °C using enzyme concentrations ranging from 0.1 nM to 1.0 μM and a (prewarmed) suspension of chloroform- or buffer-treated cells at an OD450 of ∼1.0, unless otherwise stated. Lysozyme from hen egg white (Roche; 1.0 μM final concentration) and 50 mM Tris-HCl pH 8.0 were routinely used as positive and negative controls, respectively. For the determination of pH optimum, the appropriate pH in the reaction mixtures was obtained using Britton–Robinson buffer^63^ adjusted to a pH ranging from 5.0 to 10.0 (final concentration 0.1 M).

### Crystallization, Diffraction Data Collection, Structure Determination, and Model Refinement

An initial hit with the full-length *Pa*GH19Lys protein with a C-terminal His tag was identified with the JCSG plus MD1–37 kit using 24-well VDE crystallization plates (Hampton Research) with the hanging-drop vapor-diffusion technique. The final and optimized reservoir solution used for growing crystals consisted of a solution of 0.1 M Tri-Sodium Citrate dihydrate, pH 5.0/5.5, and 15% PEG3350. 1.2 uL of protein (10 and 15 mg/mL) was mixed with 1.2 uL of reservoir solution and incubated at room temperature. Crystals were readily visible after 1–2 days of incubation.

Diffraction data were collected at the ID30B beamline at the European Synchrotron Radiation Facility (ESRF, Grenoble, France). X-ray data were processed using the EDNA autoprocessing pipeline^64^ at ESRF, including programs like indexing and integrating data (XDS^65^), and scaling, merging, and truncating integrated data (POINTLESS,^66^ AIMLESS,^67^ and TRUNCATE^68^). The structure was solved by the molecular replacement program PHASER^69^ within the PHENIX^70^ program package, using the Alphafold v2.0 predicted structure of UniProt ID: A0A0H2ZLP8 as a search model.^71^ The structure was refined using phenix.refine^72^ within the PHENIX suite, and model manipulations were performed in COOT.^73^ Iterative cycles of positional refinement in PHENIX, interspersed with manual rebuilding in COOT, were carried out until residues possessed well-defined electron density and no further improvements of the Rwork and Rfree factors were observed. Data collection and refinement statistics are summarized in Table 3. Structure factors and coordinates have been deposited in the Protein Data Bank (PDB accession number: 9EOI). Molecular graphics were rendered using PyMOL (PyMOL Molecular Graphics System, Version 1.20, Schrödinger, LLC).

**Table 3:** Data Collection and Refinement Statistics for *Pa*GH19Lys (PDB Accession Number: 9EOI)

**data collection**		**refinement statistics**	
beamline	ID30B (ESRF, Grenoble)	*R*_work_^b^/*R*_free_^c^	0.216/0.254
wavelength (Å)	0.8731	macromolecules/a.s.u.	2
space group	P 21 21 21	protein	418
cell parameters – *a*, *b*, *c* (Å)	65.41, 79.70, 91.61	solvent	267
cell parameters – α, β, γ (°)	90.00, 90.00, 90.00	ligands	2
resolution (Å)	45.808–1.770 (1.833–1.770)^a^	r.m.s.d. from ideal values	
number of unique reflections	47293 (4592)^a^	bond lengths (Å)	0.0065
multiplicity	2.0 (2.0)^a^	bond angles (°)	0.87
completeness (%)	99.9 (99.2)^a^	Ramachandran plot	
mean *I*/σ(*I*)	10.1 (1.1)^a^	most favored (%)	98.55
*R*_meas_	0.051 (0.87)^a^	allowed (%)	1.45
Wilson B-factor (Å^2^)	26.66	outliers (%)	0

a Values in parentheses are for the outer shell (high-res.).

b $R_{work}=\sum (|F_{obs}-F_{calc}|)/\sum |F_{obs}|\;$

c *R*_free_ is calculated from a randomly chosen 5% sample of all unique reflections not used in the refinement.

### Purification of Peptidoglycan from UCBPP-PA14

Cultures of UCBPP-PA14 were prepared by growing the bacteria in triplicate in LB medium overnight at 37 °C with shaking (225 rpm). Overnight cultures were diluted 1:50 in 2 L of LB medium and grown at 37 °C with shaking (225 rpm). After approximately 6 h, corresponding to the exponential growth phase of the bacteria, the cells were harvested by centrifugation at 14,000*g* for 15 min, and flash frozen in liquid nitrogen. To lyse the cells, the frozen pellets were resuspended in 30 mL of boiling double-distilled water, and SDS was added to a final concentration of 5%. Next, the bacterial-SDS suspensions were boiled for 45 min with stirring and left to cool down to room temperature. Suspensions were then pelleted by centrifugation at 215,000*g* for 2 h at 20 °C (Ti45 rotor, Beckman). The supernatants were discarded, and the pellets were washed and resuspended in 70 mL of double-distilled water, followed by centrifugation. Again, the supernatants were discarded, and the procedure was repeated until no SDS was visible in the solution. The thoroughly washed pellets were resuspended in 1 mL of 50 mM Tris-HCl at pH 8.0, and Trypsin (Sigma) was added to a final concentration of 0.3 mg/mL to remove protein. The reaction was incubated for 8 h at 37 °C with shaking (200 rpm). Trypsin was inactivated by boiling the solution for 30 min in 1% SDS. After cooling down the samples to room temperature, the insoluble material left in the suspension, i.e., pure peptidoglycan, was collected by centrifugation at 365,000*g* (MLA-80 rotor, Beckman). SDS was removed by repeated washes with double-distilled water by centrifugation. The final product was freeze-dried and stored at −20 °C until further use.

### Peptidoglycan Product Analysis Using UPLC-MS/MS

Purified PG (1 mg) was digested in reaction mixtures (600 μL) containing 20 mM Tris-HCl buffer at pH 7.5 and 20 μM *Pa*GH19Lys or 50 units Mutanolysin (Sigma). The reactions were incubated at 37 °C with shaking at 800 rpm for 24 h in a Thermomixer C (Eppendorf, Hamburg, Germany). All reactions were run in triplicate. After incubation, the reactions were terminated by heating at 100 °C for 5 min. The samples were then centrifuged at 16,900*g* for 5 min (Eppendorf 5418R centrifuge) to sediment insoluble material and precipitated protein. 50 μL supernatant of the reactions, containing the soluble muropeptide products, was transferred and adjusted to 125 mM Borat buffer pH 9.2, yielding a final volume of 100 μL. To reduce the muropeptides in the sample, 1 mg of sodium borohydride was added to the tubes, dissolved by vortexing, and followed by incubation for 20–30 min at room temperature. Afterward, the pH of the samples was adjusted to an acidic pH (3–4) using 25% orthophosphoric acid. Samples were desalted and purified, using BioSPE PurePep Broad C18 SPE spintips (Affinisep) in combination with the Supelclean ENVI-Carb material (Sigma, catalog number 57210-U). In more detail, the spintips were prepared by pipetting 1.5 mg of the Supelclean ENVI-Carb slurry into the premade BioSPE PurePep Broad C18 SPE tips. The tips were then centrifuged at 1500*g* for 2 min, discarding the flowthrough. For purifying the samples, the spin tips were first conditioned twice using 100% acetonitrile before being conditioned twice using 0.5% trifluoroacetic acid. During both steps, the spin tips were centrifuged at 1500*g* for 2 min, discarding the flowthrough. Next, 50 μL of the digested samples were added to the spin tips and centrifuged, discarding the flowthrough. The C18 material, now containing the bound muropeptides, was washed twice with 0.5% trifluoroacetic acid, centrifuging at 1500*g* for 2 min and discarding the flowthrough. The bound products were then eluted using 40 μL 70% acetonitrile in water by centrifuging at 1500*g* for 2 min. The elution step was repeated once more, and the flowthrough from both times was kept. The eluted products were then dried using a SpeedVac system until complete dryness and finally redissolved by suspending the material in 25 μL 0.1% formic acid, followed by sonication in a water bath for 10 min to fully dissolve the sample.

MS analysis of the reduced muropeptides was performed by injecting 10 ng of the products (in direct injection mode) into an Ultimate 3000 nano UPLC (Thermo Fisher) connected to a Q-Exactive hybrid quadrupole–orbitrap mass spectrometer (Thermo Fisher) equipped with a nanoelectrospray ion source. Separation was achieved using a nanoviper Acclaim PepMap 100 C18 column (50 cm × 75 μm) (Thermo Fisher) at 50 °C with an isocratic flow of 0.3 μL/min. The analytes were separated using a 55 min gradient of solvent A (water, 0.1% [v/v] formic acid) and solvent B (acetonitrile, 0.1% [v/v] formic acid): 4–8% B for 8 min, 8.0–9.5% B for 8 min, 9.5–12.5% B for 9 min, 12.5–30% B for 10 min, followed by washing for 5 min with 80% B, and re-equilibration with 4% B for 15 min.

The Q-Exactive hybrid quadrupole–orbitrap mass spectrometer (Thermo Fisher) was operated in positive-ion-data-dependent acquisition mode. The full scan acquisition mass range was set to 150–2250 *m*/*z* with a resolution of 70,000 and an automatic gain control (AGC) target of 1 × 10^6^ ions. Data-dependent MS/MS was applied, fragmenting the three most intense ions at any given time. The MS/MS settings were as follows: resolution of 17,500, AGC of 1 × 10^5^ ions; maximum IT of 50 ms; dynamic exclusion time of 20 s; and higher-energy collisional dissociation energy of 28.

### MS Data Deconvolution and Analysis

For deconvolution and analysis, both Protein Metrics Byos (version 5.2.31) and PGfinder (version 1.2.0) were used as described previously.^42^ PGfinder (version 1.2.0) was mainly used for identification and quantification,^42^ while Protein Metrics Byos (version 5.2.31) was used for deconvolution and MS/MS analysis of the PG structures for verification. For PGfinder (version 1.2.0), the simple mass database was used for matching and identification of PG monomer structures.^42^ The ppm tolerance was set to 5, the cleanup window was set to 0.5 min, and acetylation, deacetylation, and anhydro were set as modifications. Structures having retention times after 35 min were not included in the final analysis. The average intensities, retention times, observed monoisotopic masses, and parts per million differences were calculated by consolidating the data from the individual matching outputs.

For a comparison of the abundances of the different monomeric PG structures between *Pa*GH19Lys and mutanolysin, the intensity values were log_2_ transformed. Only matched PG monomers that had intensity values for all three replicates for each enzyme and were verified by MS/MS were included in the analysis. Statistically significant differences in the abundance of individual structures were determined through the performance of multiple unpaired Student’s *t* tests, with a significance level of p = 0.05 and a false discovery rate (FDR) of p = 0.01. PG monomers were considered to have a significant difference in abundance if their log_2_ fold-change values were ± 0.58 (fold change of ± 1.5).

## Supplementary Material





## Data Availability

The MS data obtained in this study can be accessed in the GlycoPOST data repository (https://glycopost.glycosmos.org/) using the accession number: GPST000448.

## Notes

Supplementary Figures S1–S22 including sequence alignment of putative spanins, *Pa*GH19Lys activity against chitin, chitohexaose, and chitosan, activity of *Pa*GH19Lys at 4 °C, UHPLC-MS chromatograms of digested peptidoglycan, and annotated MS2 spectra of monomers (PDF)
Supplementary Table S1 PG monomer product profiles with and without modifications for *Pa*GH19Lys and mutanolysin (XLSX)

## Notes

*Pa*GH19Lys: A0A0H2ZLP8 (UniProt).
