# Supplementary material for: Cryptic Prophage Endolysin Is a Highly Active Muramidase
Source: Biochemistry. 2025 Jul 9;64(15):3446–58. doi: 10.1021/acs.biochem.5c00142 (PMC12329726; doi:10.1021/acs.biochem.5c00142)
Supplement: Supplementary file 1 [file bi5c00142_si_001.pdf]

Supporting information

The *Pseudomonas aeruginosa* cryptic prophage endolysin is a highly active muramidase

Per Kristian Thorén Edvardsen[1], Andrea Nikoline Englund[1], Åsmund Kjendseth Røhr[1], Stéphane Mesnage[2], and Gustav Vaaje-Kolstad[1*]

[1]Faculty of Chemistry, Biotechnology and Food Science, Norwegian University of Life Sciences, 1432 Ås, Norway.

[2]School of Biosciences, University of Sheffield, Sheffield S10 2TN, United Kingdom.

*Corresponding author; gustav.vaaje-kolstad@nmbu.no

Keywords: *Pseudomonas aeruginosa*, GH19, lytic enzyme, endolysin, muramidase, peptidoglycan.

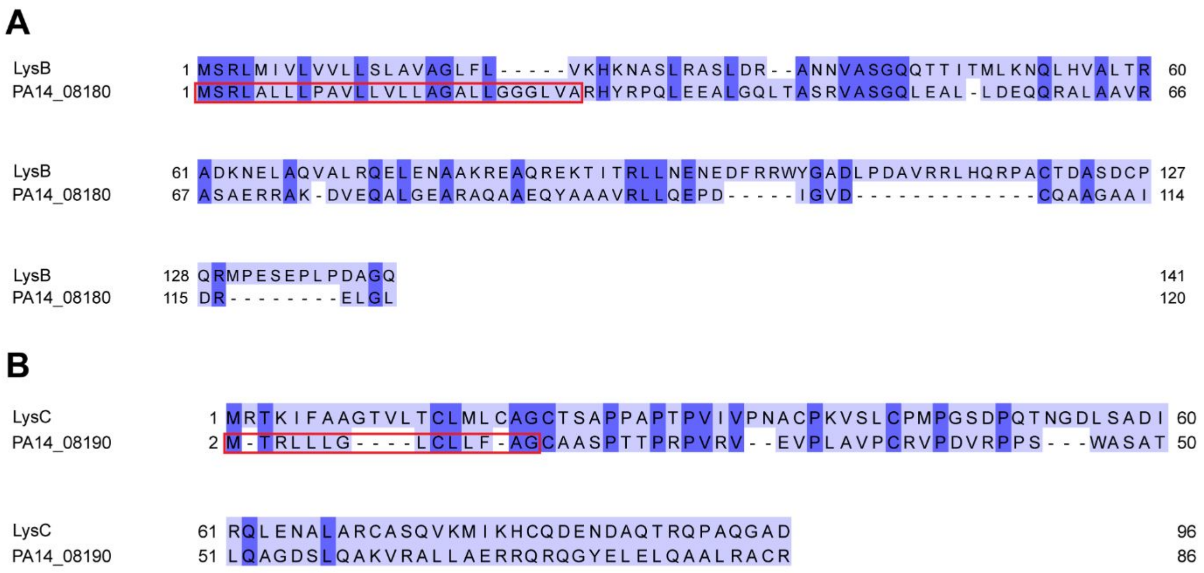

Supplementary Figure 1. Sequence alignment of putative spanins. (A, B) Sequence alignment of PA14_08180 and PA14_08190 with the LysB (Uniprot ID: P51770) and LysC (Uniprot ID: Q8LTD0) spanins using MUSCLE with default settings in Jalview [1]. The signal peptides are indicated by red squares.

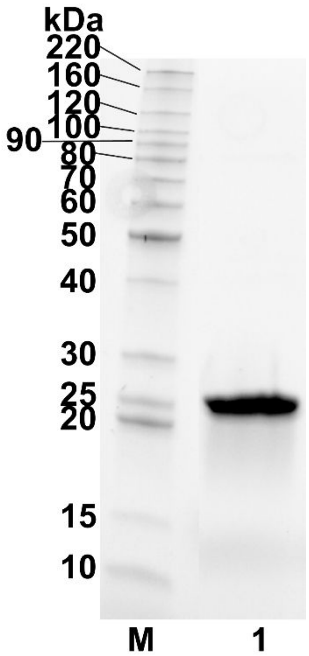

Supplementary Figure 2. SDS-PAGE analysis of purified *Pa*GH19Lys from *E. coli* C43. Lane 1, standard molecular weight markers (kDa); lane 2, *Pa*GH19Lys.

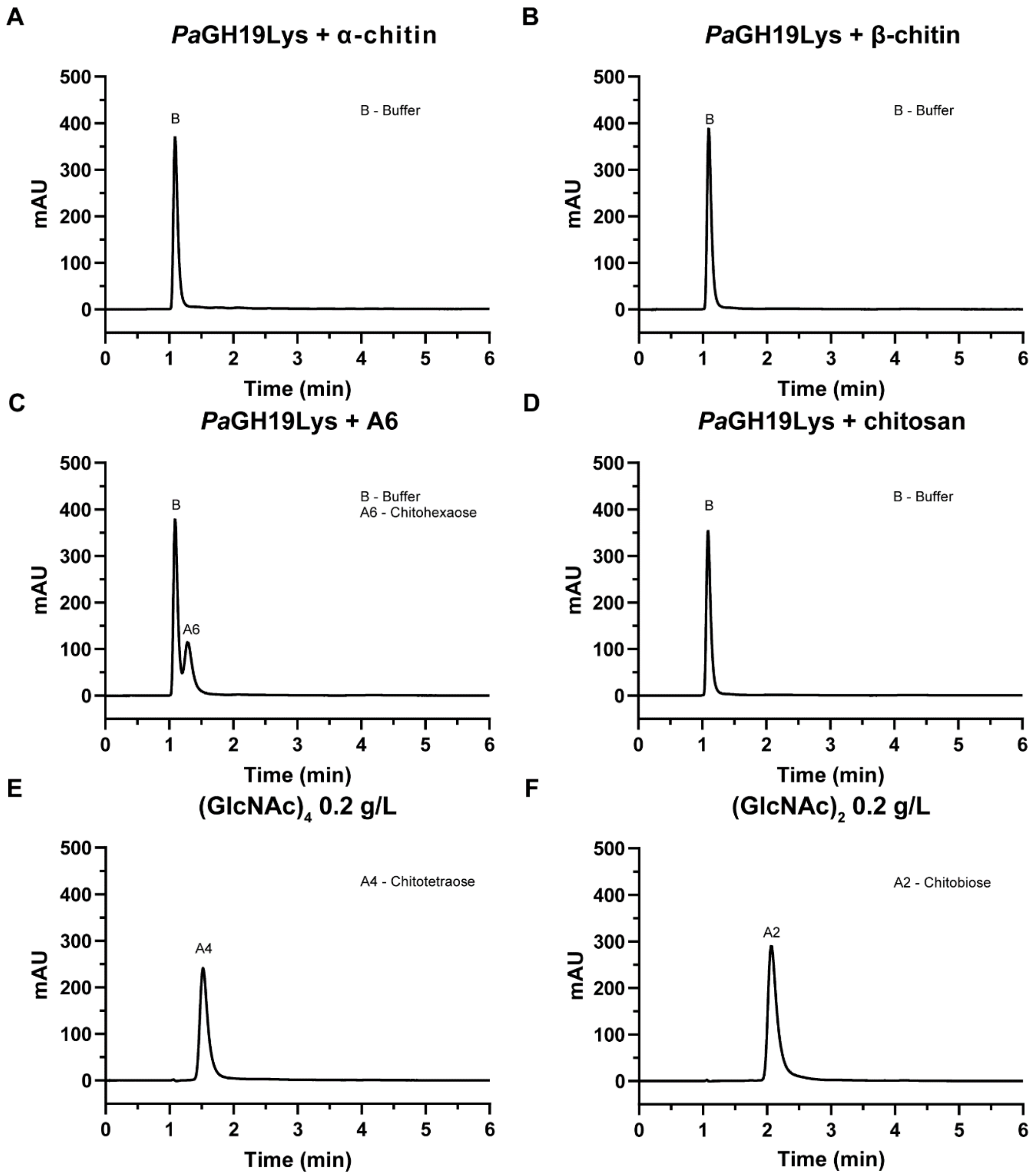

Supplementary Figure 3. *Pa*GH19Lys activity against chitin, chitohexaose and chitosan. Chromatograms showing product formation after incubation of *Pa*GH19Lys with (A) α- chitin, (B) β-chitin (right), (C) chitohexaose, and (D) chitosan for 2 hours at 37 ℃, pH 8.0. The sugar standards of (E) 0.2 g/L chitotetraose, and (F) 0.2 g/L chitobiose were also included as controls. The UV was measured at a wavelength of 194 nm.

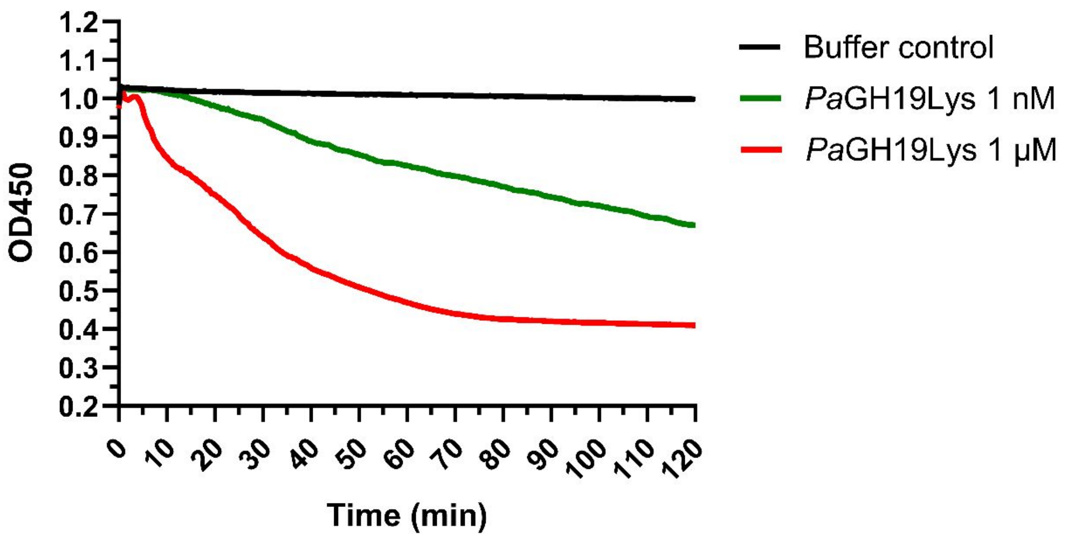

Supplementary Figure 4. The activity of *Pa*GH19Lys against *P. aeruginosa* sacculi with 1 nM and 1 µM of enzyme incubated at 4 °C. The data are plotted as the mean, representing four experiments.

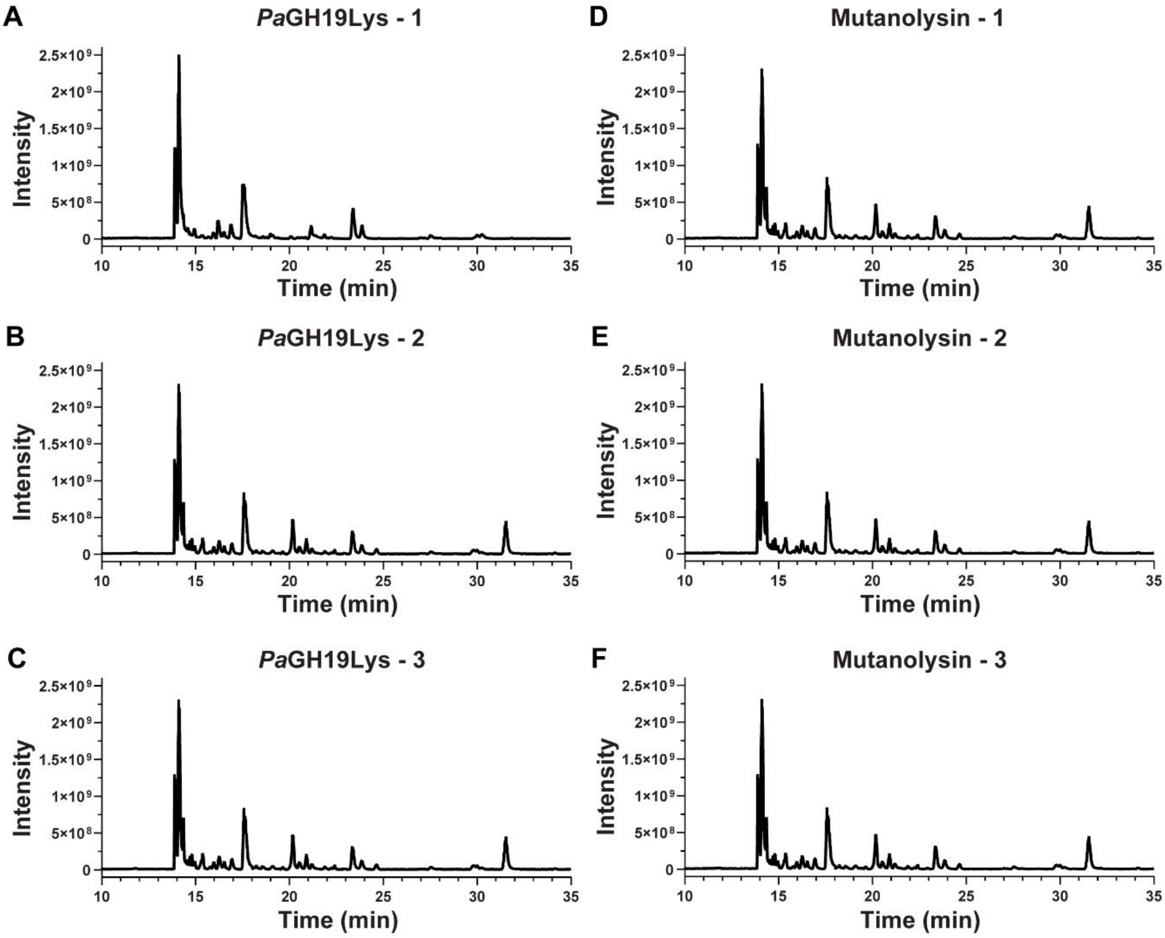

Supplementary Figure 5. UHPLC-MS chromatograms of *P. aeruginosa* digested peptidoglycan using (A-C) *Pa*GH19Lys and (D-F) Mutanolysin.

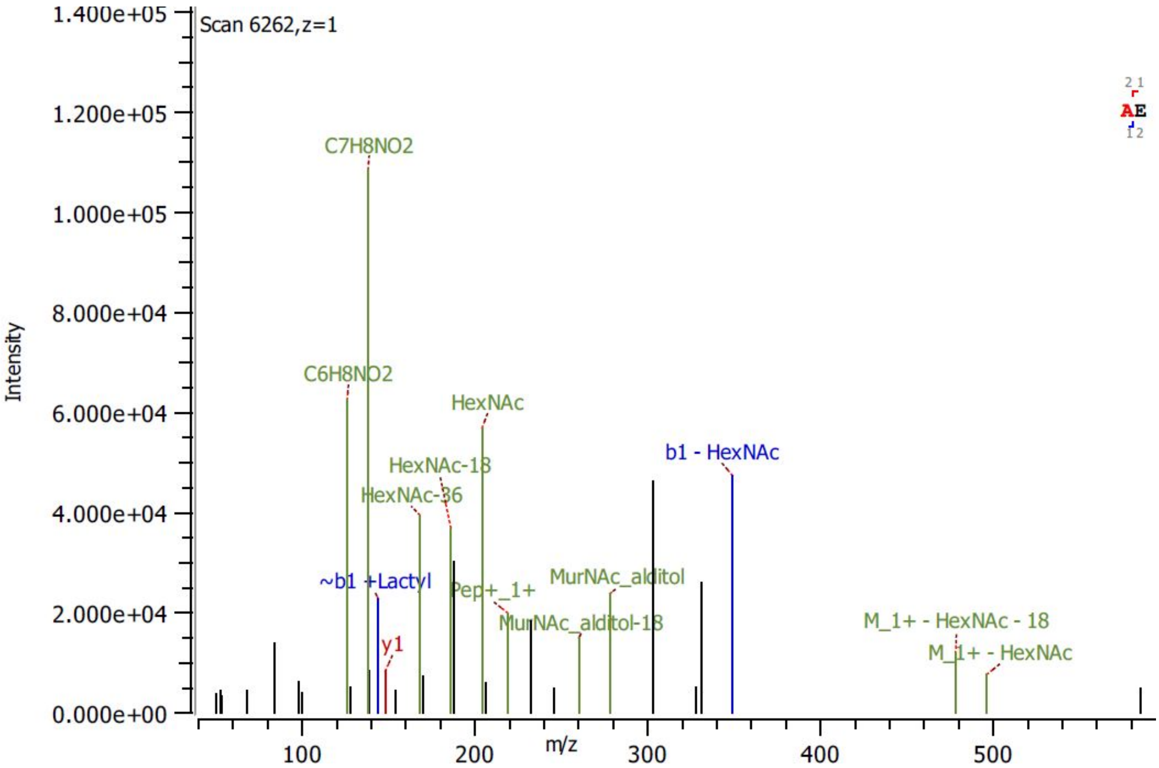

Supplementary Figure 6. Example of an MS2 annotated spectrum of gm-AE. The Byonic module from Byos was used to annotate and identify the muropeptide. The software was prompted to searched for disaccharides (GlcNAc-MurNAc(gm)) containing mono-, di-, tri-, tetra- and pentapeptides stems.

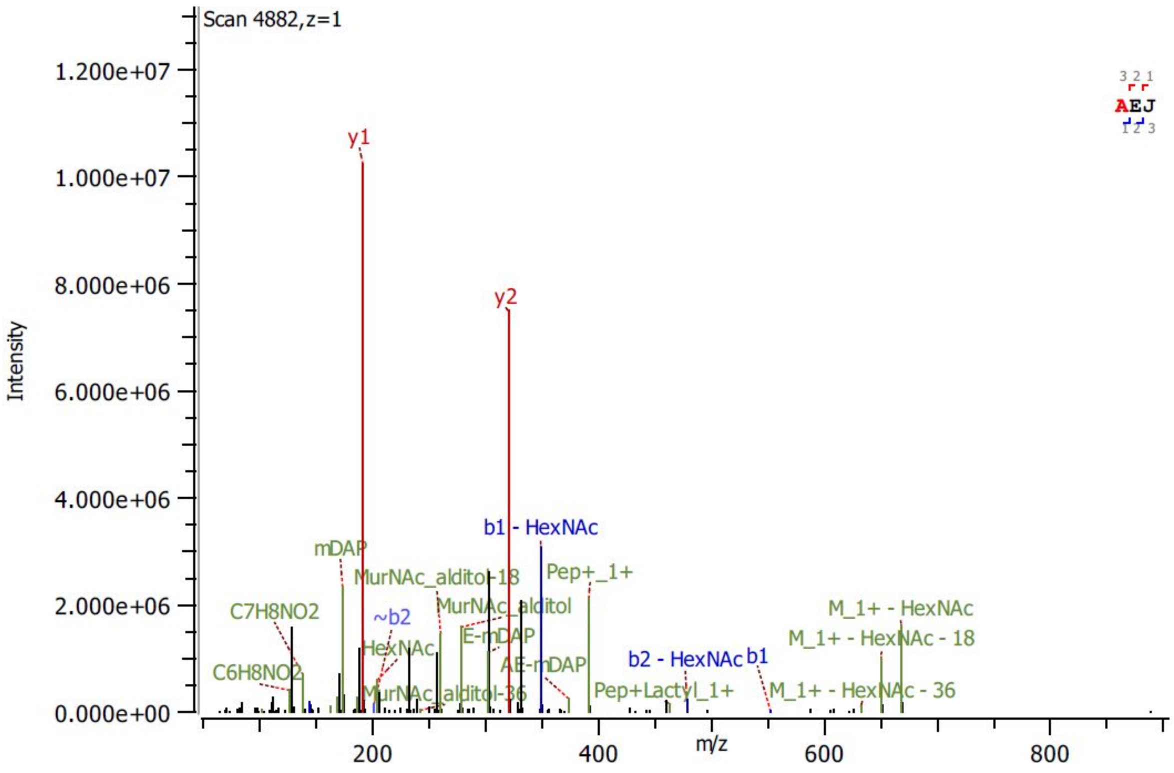

Supplementary Figure 7. Example of an MS2 annotated spectrum of gm-AEJ. The Byonic module from Byos was used to annotate and identify the muropeptide. The software was prompted to searched for disaccharides (GlcNAc-MurNAc(gm)) containing mono-, di-, tri-, tetra- and pentapeptides stems.

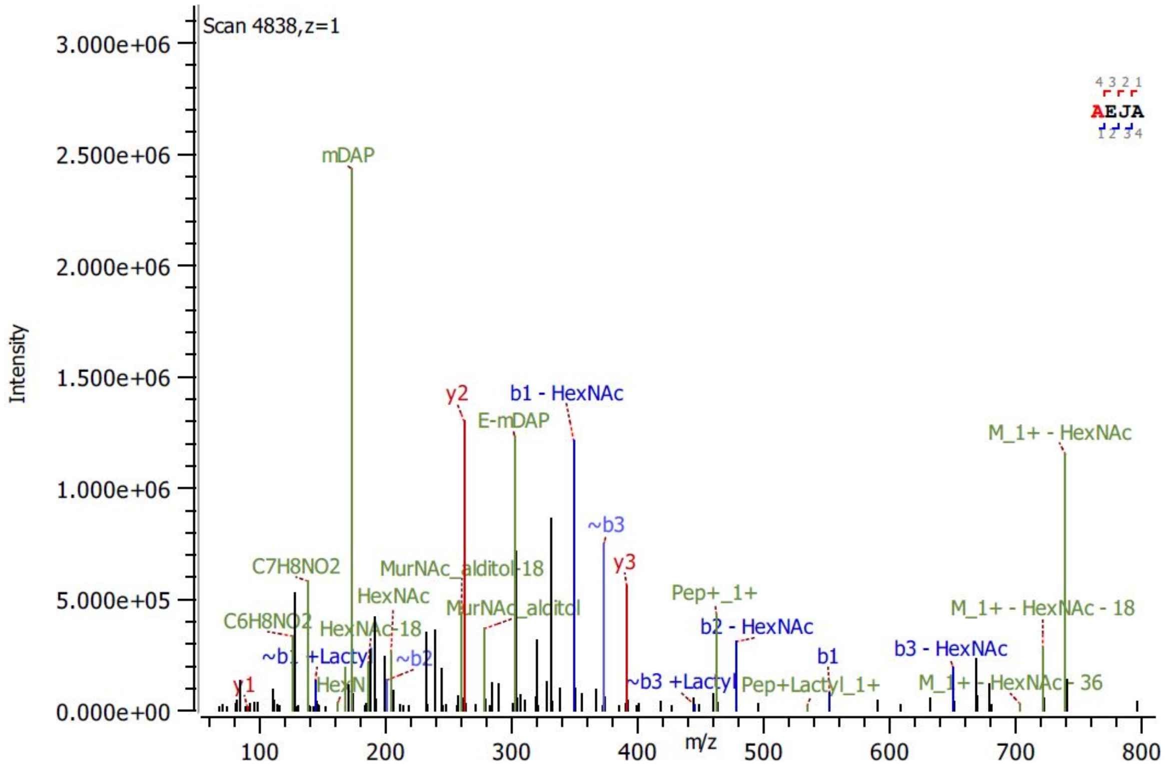

Supplementary Figure 8. Example of an MS2 annotated spectrum of gm-AEJA. The Byonic module from Byos was used to annotate and identify the muropeptide. The software was prompted to searched for disaccharides (GlcNAc-MurNAc(gm)) containing mono-, di-, tri-, tetra- and pentapeptides stems.

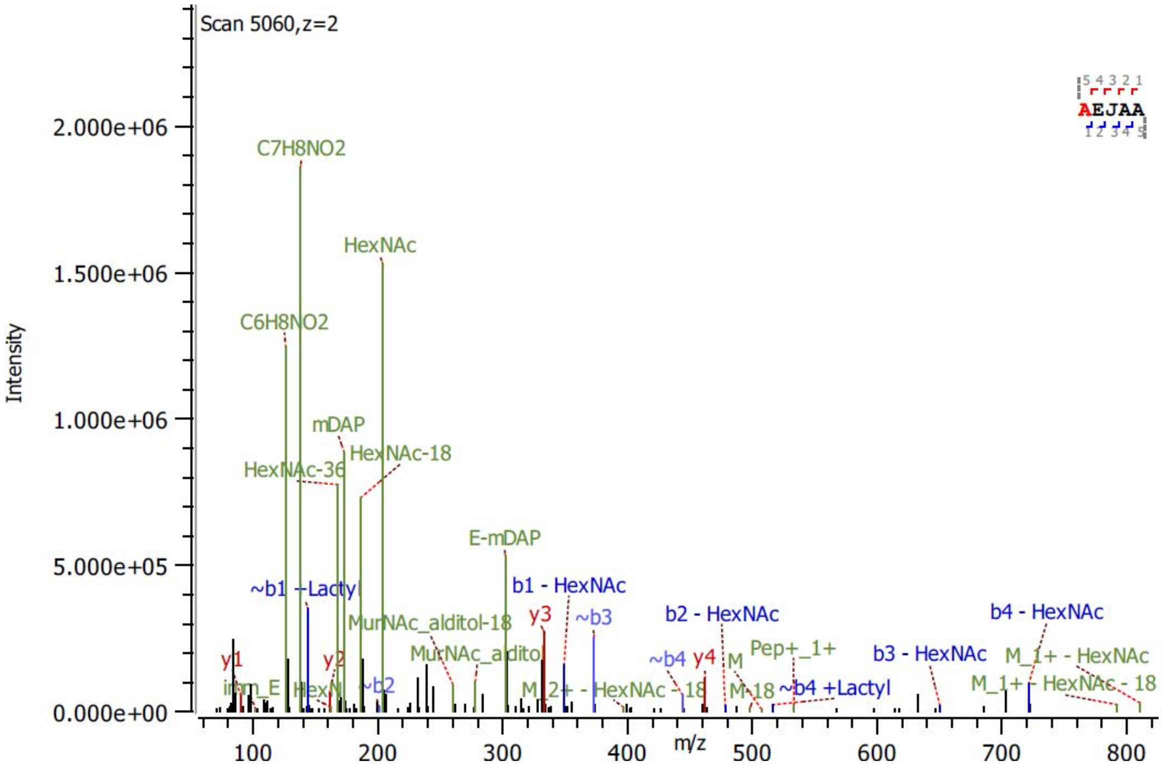

Supplementary Figure 9. Example of an MS2 annotated spectrum of gm-AEJAA. The Byonic module from Byos was used to annotate and identify the muropeptide. The software was prompted to searched for disaccharides (GlcNAc-MurNAc(gm)) containing mono-, di-, tri-, tetra- and pentapeptides stems.

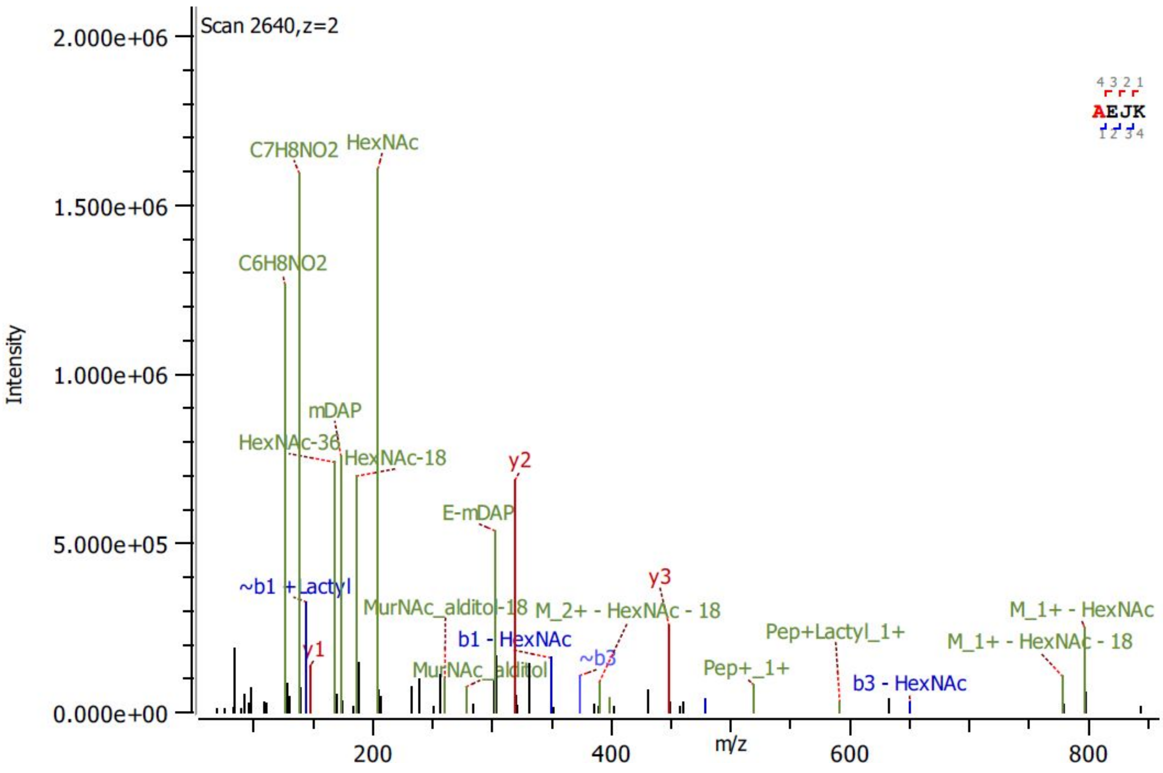

Supplementary Figure 10. Example of an MS2 annotated spectrum of gm-AEJK. The Byonic module from Byos was used to annotate and identify the muropeptide. The software was prompted to searched for disaccharides (GlcNAc-MurNAc(gm)) containing mono-, di-, tri-, tetra- and pentapeptides stems.

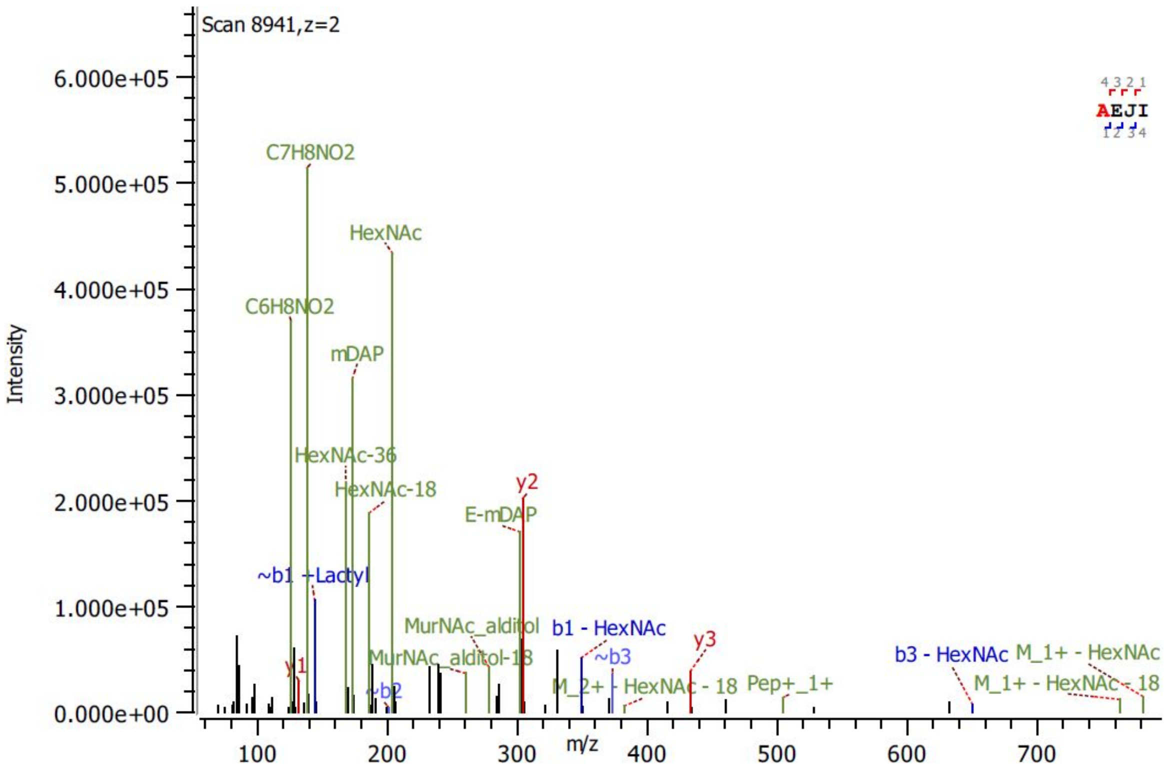

Supplementary Figure 11. Example of an MS2 annotated spectrum of gm-AEJI. The Byonic module from Byos was used to annotate and identify the muropeptide. The software was prompted to searched for disaccharides (GlcNAc-MurNAc(gm)) containing mono-, di-, tri-, tetra- and pentapeptides stems.

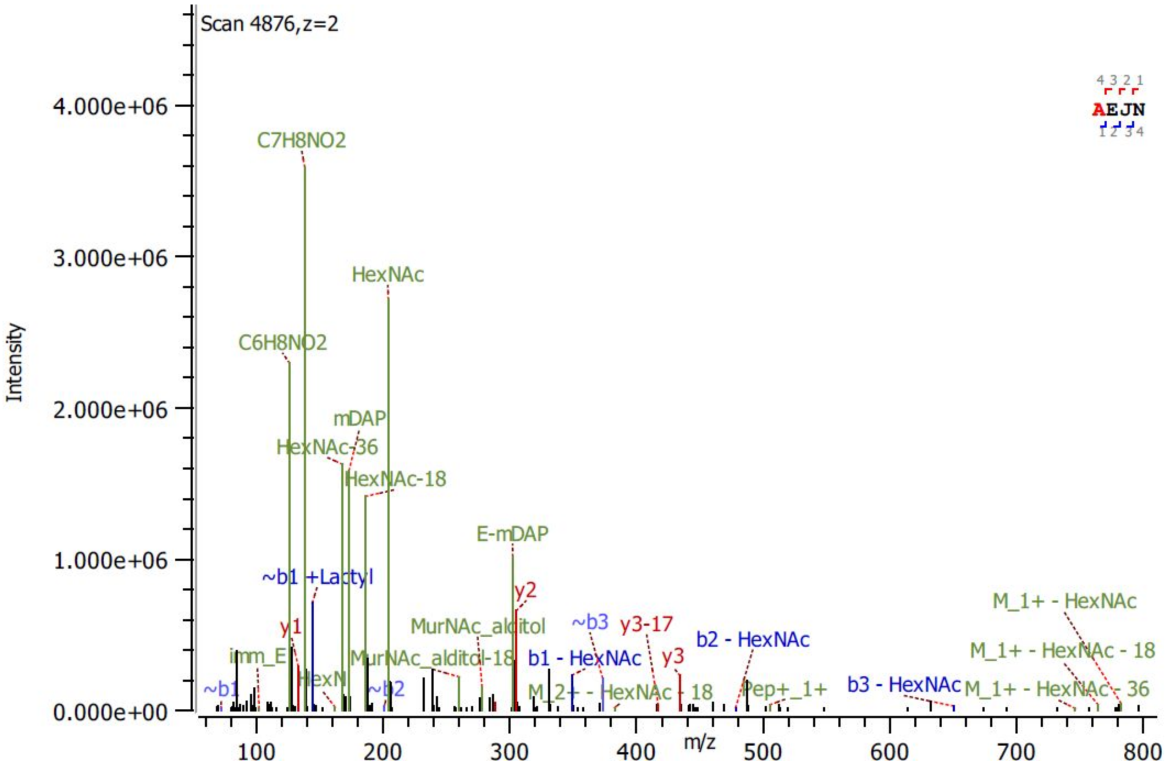

Supplementary Figure 12. Example of an MS2 annotated spectrum of gm-AEJN. The Byonic module from Byos was used to annotate and identify the muropeptide. The software was prompted to searched for disaccharides (GlcNAc-MurNAc(gm)) containing mono-, di-, tri-, tetra- and pentapeptides stems.

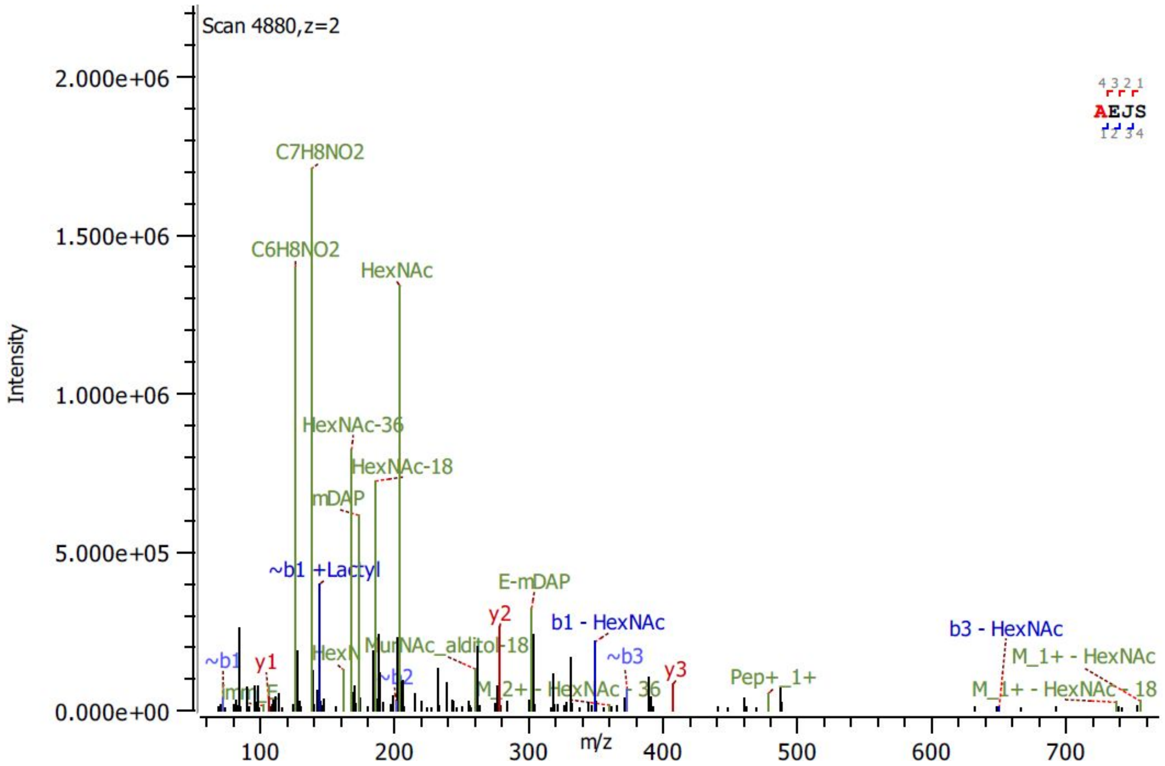

Supplementary Figure 13. Example of an MS2 annotated spectrum of gm-AEJS. The Byonic module from Byos was used to annotate and identify the muropeptide. The software was prompted to searched for disaccharides (GlcNAc-MurNAc(gm)) containing mono-, di-, tri-, tetra- and pentapeptides stems.

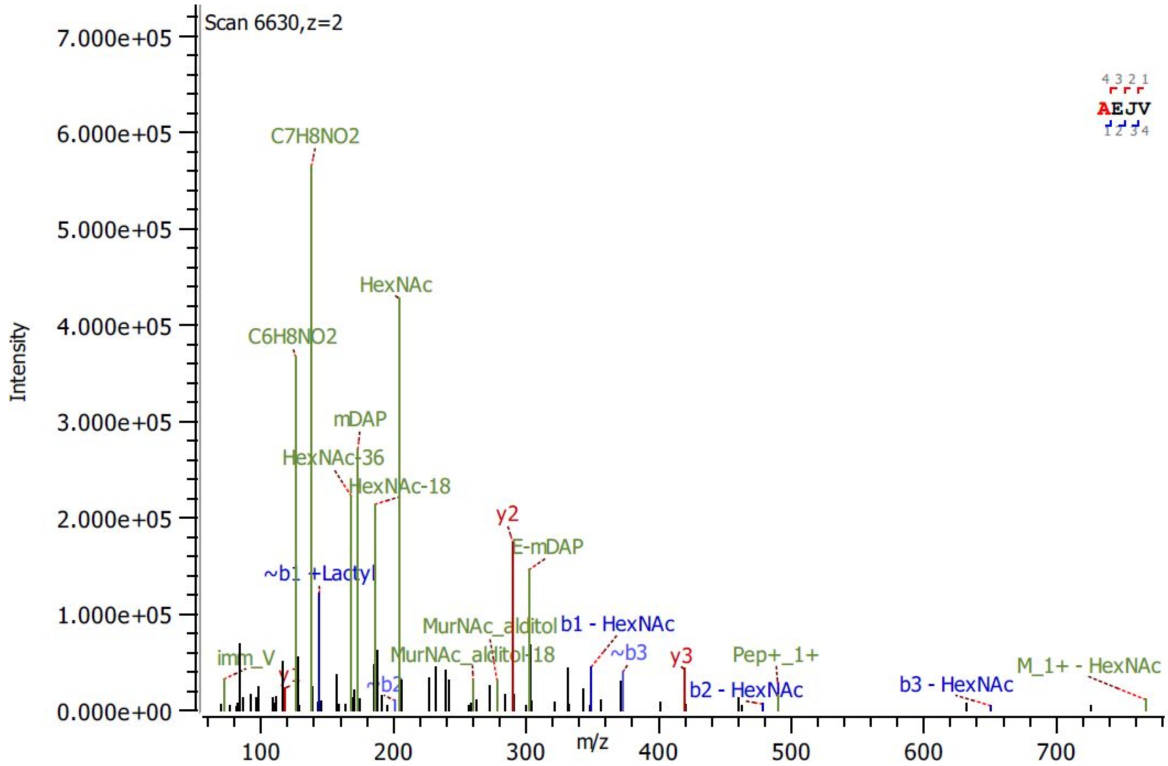

Supplementary Figure 14. Example of an MS2 annotated spectrum of gm-AEJV. The Byonic module from Byos was used to annotate and identify the muropeptide. The software was prompted to searched for disaccharides (GlcNAc-MurNAc(gm)) containing mono-, di-, tri-, tetra- and pentapeptides stems.

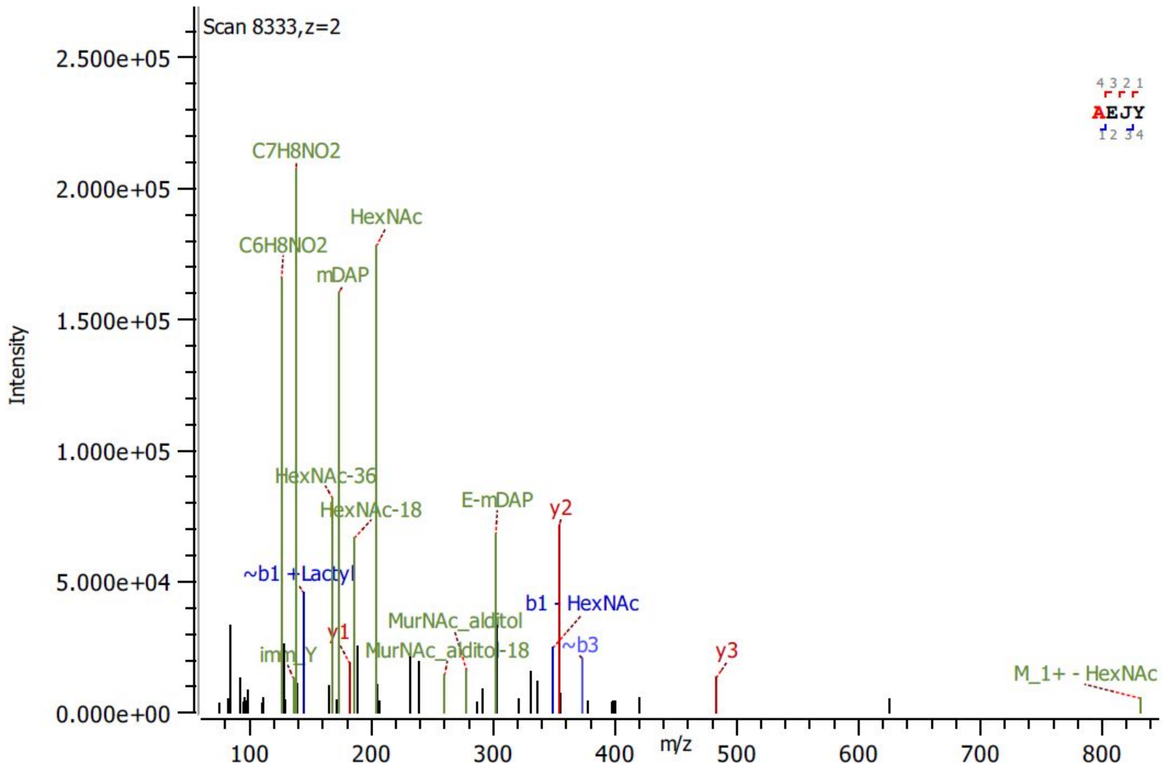

Supplementary Figure 15. Example of an MS2 annotated spectrum of gm-AEJY. The Byonic module from Byos was used to annotate and identify the muropeptide. The software was prompted to searched for disaccharides (GlcNAc-MurNAc(gm)) containing mono-, di-, tri-, tetra- and pentapeptides stems.

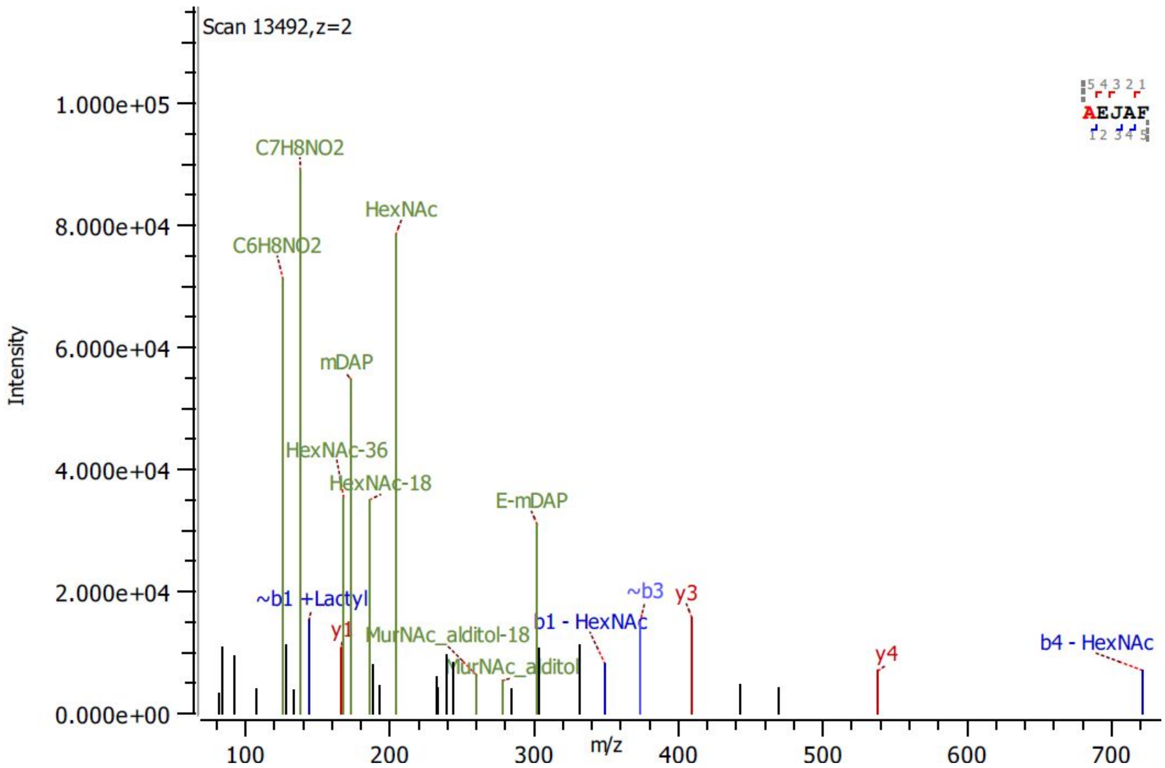

Supplementary Figure 16. Example of an MS2 annotated spectrum of gm-AEJAF. The Byonic module from Byos was used to annotate and identify the muropeptide. The software was prompted to searched for disaccharides (GlcNAc-MurNAc(gm)) containing mono-, di-, tri-, tetra- and pentapeptides stems.

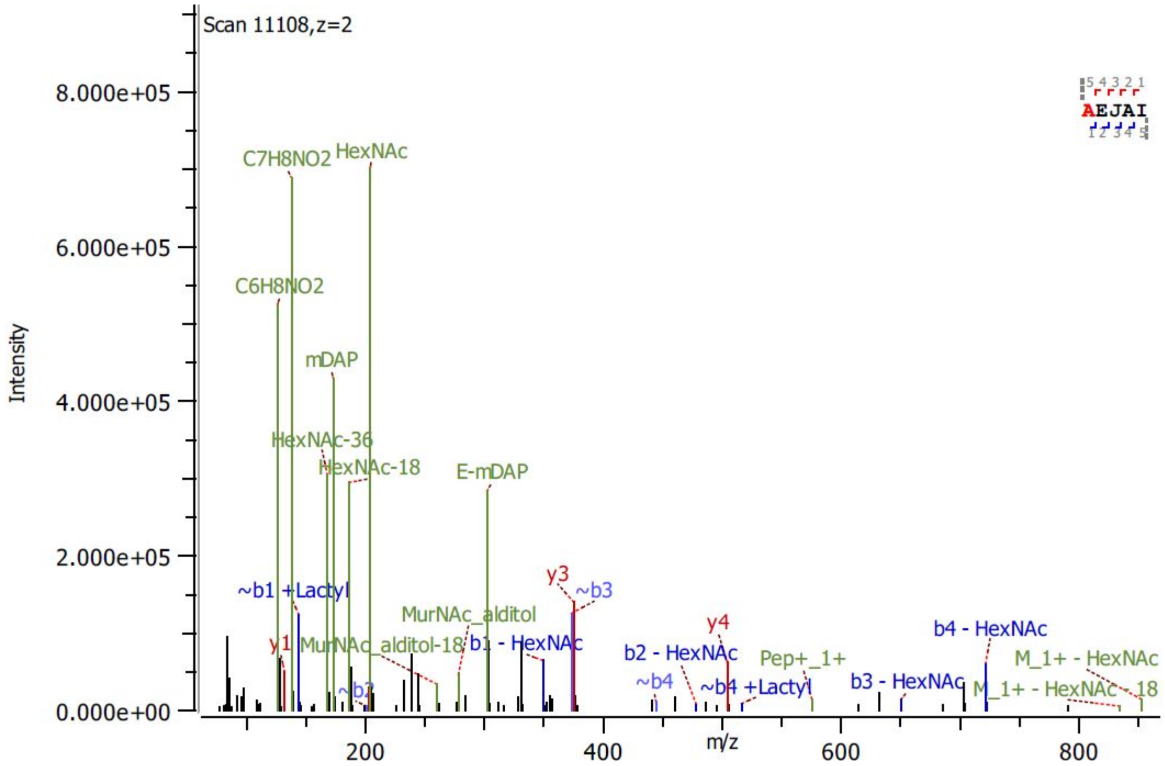

Supplementary Figure 17. Example of an MS2 annotated spectrum of gm-AEJAI. The Byonic module from Byos was used to annotate and identify the muropeptide. The software was prompted to searched for disaccharides (GlcNAc-MurNAc(gm)) containing mono-, di-, tri-, tetra- and pentapeptides stems.

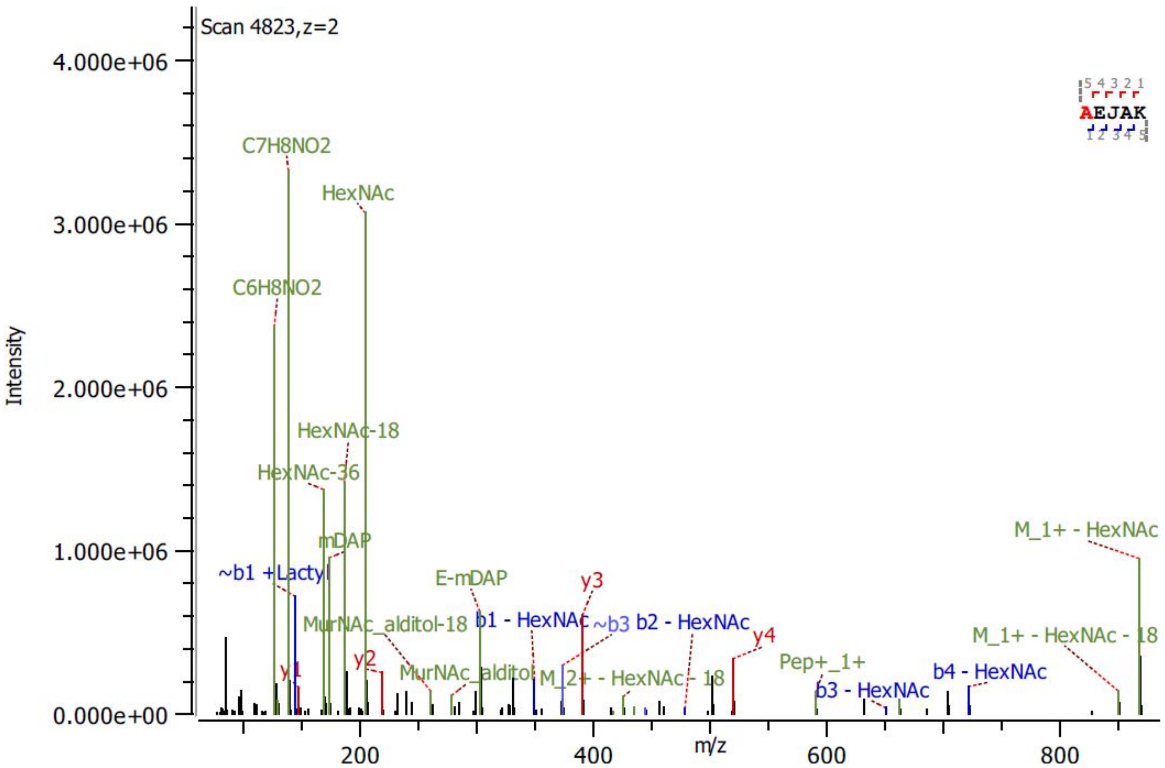

Supplementary Figure 18. Example of an MS2 annotated spectrum of gm-AEJAK. The Byonic module from Byos was used to annotate and identify the muropeptide. The software was prompted to searched for disaccharides (GlcNAc-MurNAc(gm)) containing mono-, di-, tri-, tetra- and pentapeptides stems.

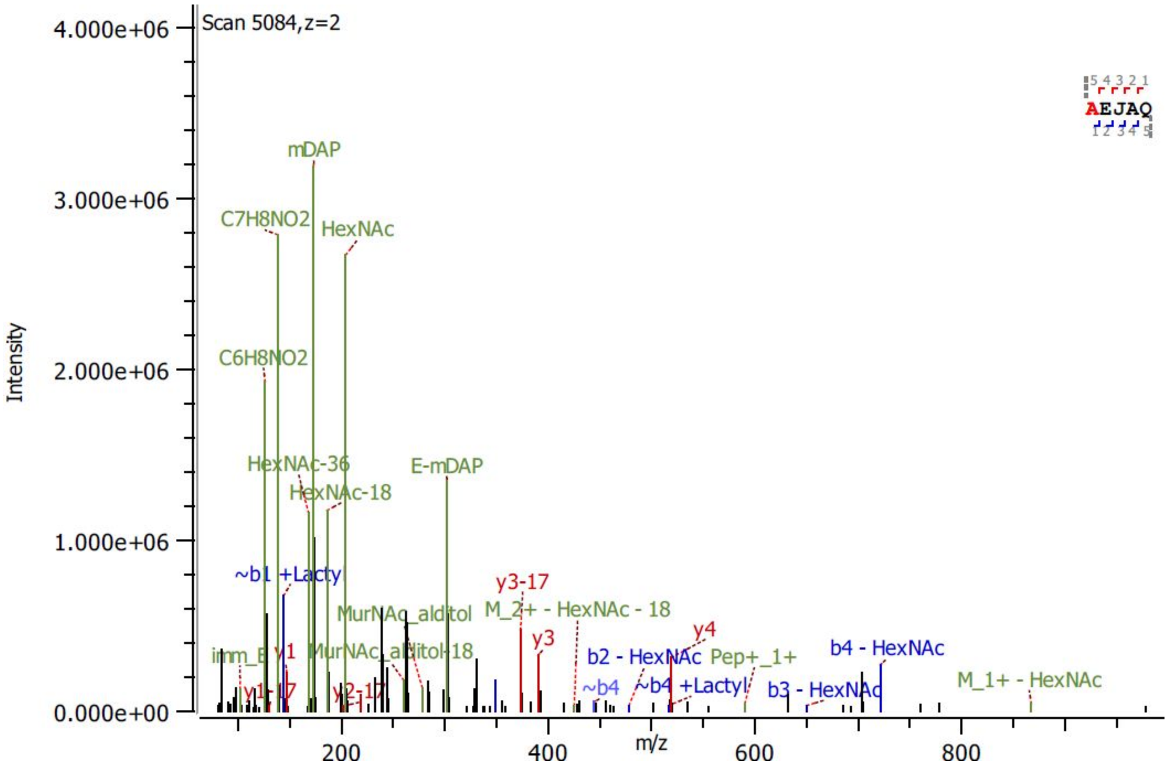

Supplementary Figure 19. Example of an MS2 annotated spectrum of gm-AEJAQ. The Byonic module from Byos was used to annotate and identify the muropeptide. The software was prompted to searched for disaccharides (GlcNAc-MurNAc(gm)) containing mono-, di-, tri-, tetra- and pentapeptides stems.

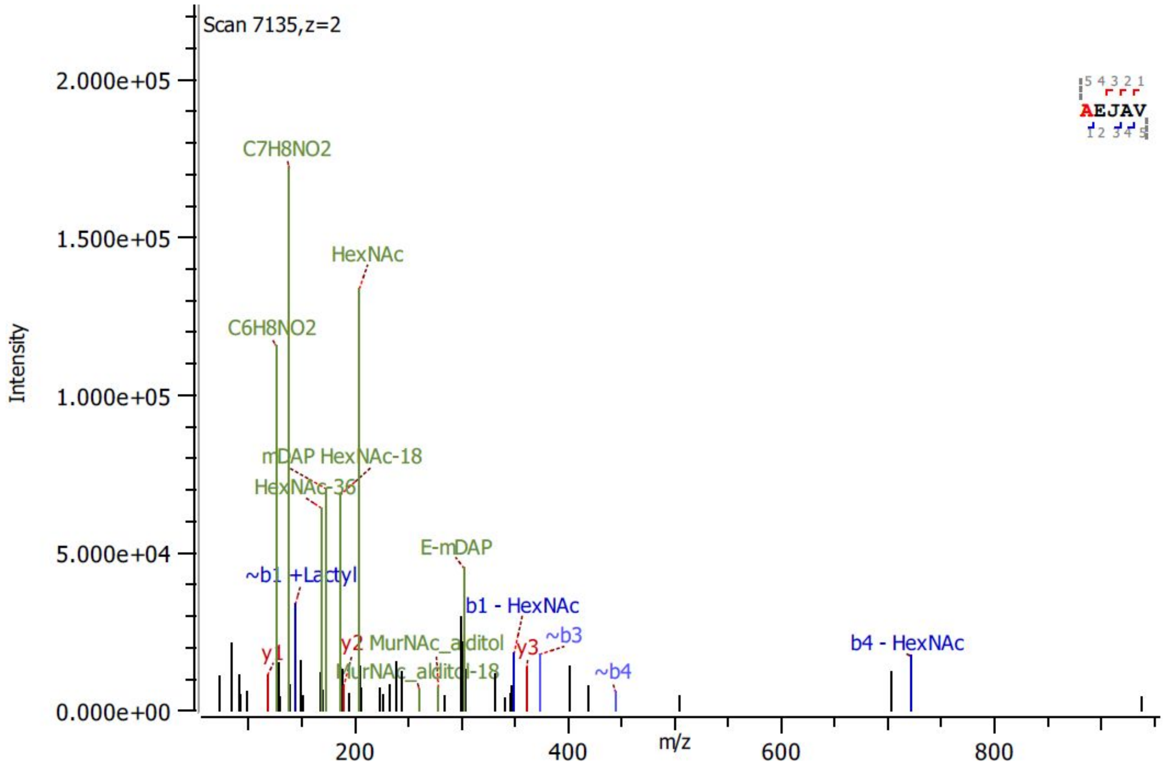

Supplementary Figure 20. Example of an MS2 annotated spectrum of gm-AEJAV. The Byonic module from Byos was used to annotate and identify the muropeptide. The software was prompted to searched for disaccharides (GlcNAc-MurNAc(gm)) containing mono-, di-, tri-, tetra- and pentapeptides stems.

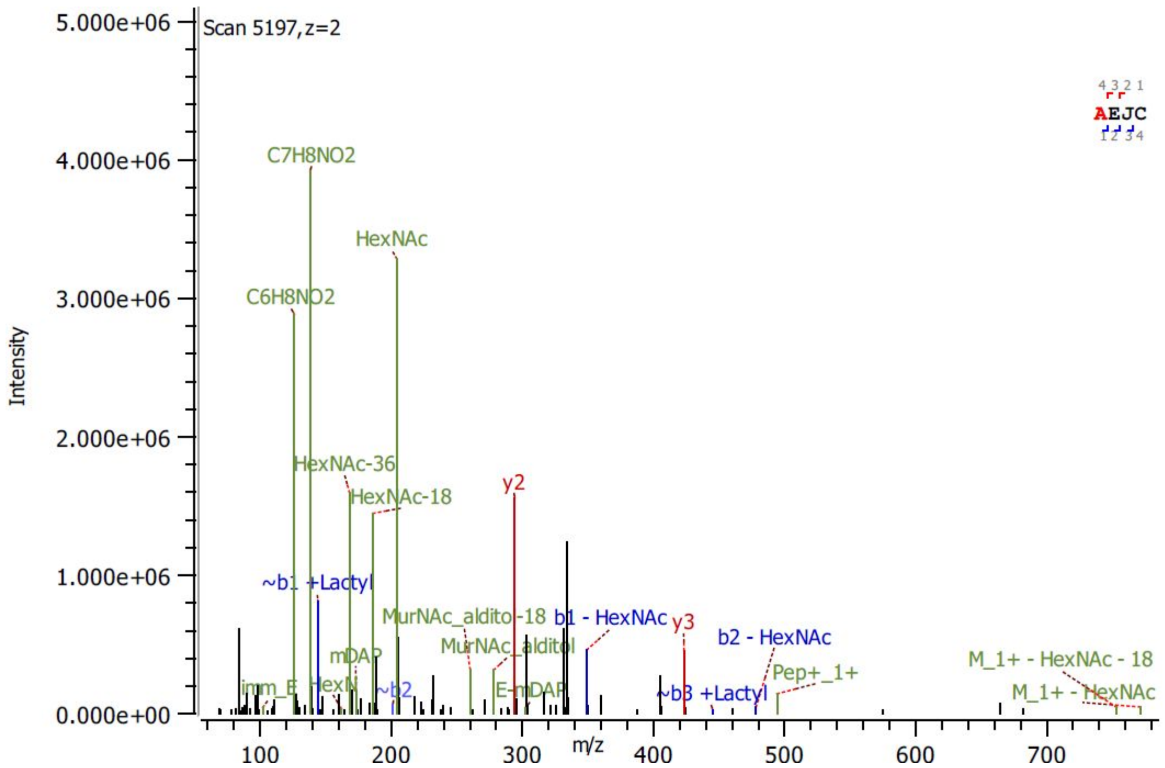

Supplementary Figure 21. Example of an MS2 annotated spectrum of gm-AEJC. The Byonic module from Byos was used to annotate and identify the muropeptide. The software was prompted to searched for disaccharides (GlcNAc-MurNAc(gm)) containing mono-, di-, tri-, tetra- and pentapeptides stems.

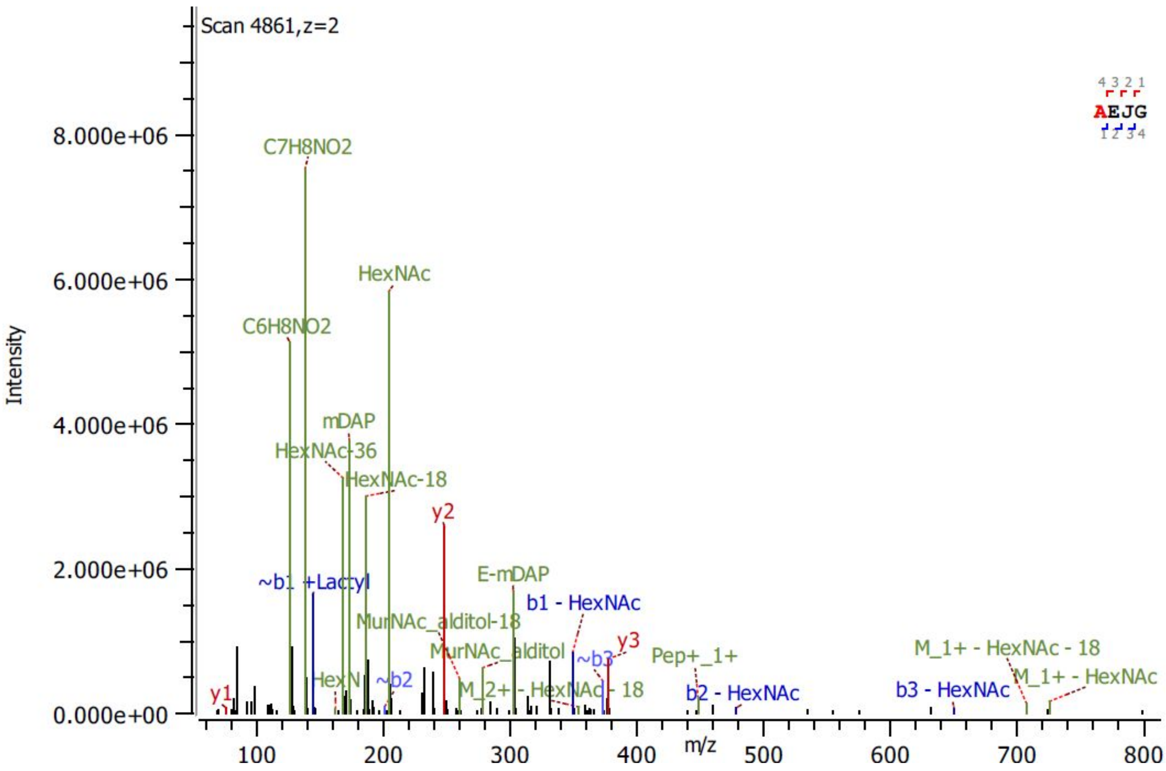

Supplementary Figure 22. Example of an MS2 annotated spectrum of gm-AEJG. The Byonic module from Byos was used to annotate and identify the muropeptide. The software was prompted to searched for disaccharides (GlcNAc-MurNAc(gm)) containing mono-, di-, tri-, tetra- and pentapeptides stems.

Supporting references

1. Waterhouse AM, Procter JB, Martin DMA, Clamp M, Barton GJ. 2009. Jalview Version 2 - A multiple sequence alignment editor and analysis workbench. Bioinformatics 25:1189–1191.
